# (Mis)perceptions of individual position in national and global income distribution. The Italian case

**DOI:** 10.3389/fsoc.2025.1646173

**Published:** 2026-01-14

**Authors:** Nevena Kulic, Olga Griaznova, Eleonora Clerici

**Affiliations:** Department of Political and Social Sciences, Università degli Studi di Pavia, Pavia, Italy

**Keywords:** misperceptions, income distribution, global inequality, center tendency bias, objective income rank, Italy, income inequality

## Abstract

This study investigates income rank (mis)perceptions in Italy, focusing on the discrepancy between individuals' subjective assessments of their income position and their objectively measured positions in national and global income distribution. The research utilizes data from a large-scale national survey Inequality between reality and perception (IneqPer, *n* = 12,000; 2024/2025). It provides consistent evidence of the pervasive central tendency bias, when individuals place themselves near to the median of income distribution regardless of their objective income rank. The findings also reveal systematic patterns of misperceptions. Lower-resource individuals tend to overestimate their income rank, while higher-resource individuals often underestimate it. Misperceptions vary significantly by gender, age, employment status, education, migration background, the type of settlement, and regional economic conditions: young, unemployed, and immigrants are more prone to overestimate their rank in income distribution, while men, older, employed, those from urban areas and more educated individuals are more likely to underestimate it. Political orientation shows no associations with misperceptions of national income rank, but left-oriented respondents are more accurate with respect to global ranks, whereas all other respondents underestimate their global rank. Regionally, residents of less affluent Southern areas tend to overestimate their income rank, whereas those in wealthier Northern regions often underestimate it. The study is one of the first that analyses both national and global income rank perceptions. The results show that central tendency bias emerges consistently across different contexts, suggesting that income distributions are broadly perceived as abstract. These insights have implications for public policy, economic behavior, and interventions addressing inequality in Italy and in the world.

## 1 Introduction

In recent years, the interplay between objective facts and subjective beliefs in shaping human behavior and socio-political attitudes has attracted increasing scholarly attention. The rise of so-called “post-factual era” (Knell and Stix, 2020, p. 1) has shifted research focus toward understanding the associations and causal relationships between subjective, often biased, beliefs and various forms of behaviors, including electoral, political, and economic decision-making (Kelly and Enns, 2010; Giugni and Grasso, 2019; Knell and Stix, 2020; Cruces et al., 2013). This trend highlights the importance of perceived realities over objective facts in influencing preferences and attitudes and apply also to the topic of inequality.

Traditional economic theories have long held an assumption that agents are rational and act based on complete and accurate information. However, contemporary perspectives challenge the classical assumption, suggesting that agents may arrive at naïve estimates for at least two main reasons. First, acquiring objective information is costly or infeasible and this leads to limited awareness of actors (Benoît and Dubra, 2011). Second, agents often fail to process all available accurate information and, consequentially, act in most cases based on bounded rationality (Cairney, 2016; Kahneman et al., 1982). Gimpelson and Treisman (2018) underscore this issue, suggesting that “most theories about political effects of inequality need to be reframed as theories about effects of *perceived* inequality” (Gimpelson and Treisman, 2018, p.1) and argue that individuals lack the conceptual tools to accurately estimate their own position.

This article aims to shed light on the way how individuals perceive their position in income distribution, both at national and global levels, compared to objective situations, and to identify the factors that correlate with these (mis)perceptions in Italy. The study has, therefore, descriptive goals, relying on a non-causal and cross-sectional design. Specifically, the study focuses on misperceived relative position in income distribution, defined as a difference between individual's self-assessed and objective income ranks, and the major correlates of these misperceptions. In the paper, we use term “(mis)perception” to emphasize the dual nature of the phenomenon under study, uniting both perceptions and biases in perceptions from objective reality. When discussing individual assessments of income ranks, we refer to them as “perceptions,” when addressing detected deviations in these assessments we use term “misperception.”

Misperceptions of these ranks may lead to systematic biases in support for policies aimed at addressing national and global inequalities, also because individuals who overestimate their real position demand less redistribution and welfare benefits and vice versa: individuals who think that they are poor(er) are more likely to demand more redistribution and welfare provision (Feichtmayer and Gründler, 2021). While a large share of existing literature has prioritized the within-country perspective and focussed on the individual position on national income distribution, the rise of globalization on the one hand, and the availability of comparative international datasets on the other hand, made it possible to compare individuals across different countries in the world based on their income and their position in the world's income distribution (Milanovic, 2016). Examining perceived income ranks within the global distribution is important because, on one hand, it typically reveals a broader range of disparities worldwide, and on the other hand, individuals often have less information about their position in the global income hierarchy compared to the income rank within their own country. Indeed, despite some decline in global interpersonal income inequality in recent years as reported by Lakner and Milanovic (2016), global inequality remains substantially higher than inequality within any individual country with World Gini points of 60 (2018, the last available year) (Milanovic, 2024). It remains unclear whether people are aware of the extent of global inequality, and whether greater uncertainty and limited information at the global level mean less accurate personal estimates of their own position within that hierarchy.

Moreover, studying (mis)perceptions of global income position matters not only because global inequality is central to “global justice” debates and equitable resource distribution among the world's citizens (Anand and Segal, 2008), but also because these preferences increasingly shape the political feasibility of such agendas. Public (mis)perceptions can either reinforce or constrain political support for global redistributive initiatives such as global taxation, international aid, and migration (Hvidberg et al., 2020; Fehr et al., 2022; Sattler-Bublitz et al., 2024).

This study has three primary objectives: to describe the distribution of perceived income ranks at both national and global levels in Italy and their deviation from the objective realities, to estimate the extent of misperceptions about individual income positions in national income distribution and global income distribution, and to identify individual and contextual correlates that are associated with them. Building on previous research that reveal constant inconsistency and biases in individual perceptions of income ranks (Norton and Ariely, 2011; Chambers et al., 2014; Cruces et al., 2013; Fernández-Albertos and Kuo, 2018; Engelhardt and Wagener, 2018; Bublitz, 2022), this study tests prior findings in a different context, and investigates whether Italians accurately position themselves in national and global income distribution, and whether this depends on their socio-demographic characteristics, their political orientation or geographical context. To what extent can these perceptions serve as reliable indicators of their actual income situation? How do these (mis)perceptions vary across different demographic groups, such as among men and women, younger and older age cohorts, individuals with different level of education and rural and urban settlements? Does political orientation play a role? Additionally, this study examines differences in perceptions across geographical contexts with different economic conditions and the level of development with Southern regions of Italy being comparatively poorer (D'Adamo et al., 2021).

To address these questions, this article utilizes novel data from a large-scale comprehensive IneqPer survey on inequality perceptions in Italy. The data were collected in the months of November and December 2024 and February and March 2025 by the survey agency Dynata. The total survey sample consists of 12,000 respondents.

The study is organized into four sections. The first section reviews the literature on individuals' perceptions of their place within both national and global income distribution, as well as correlates of these perceptions. The second section introduces the data and the methodological framework employed in the study. Next, the results section presents a descriptive analysis of perceived income distributions and multivariate regression analyses identifying the key individual and contextual correlates of misperceptions. The final discussion section interprets these findings and concludes.

## 2 Theoretical background

### 2.1 Individuals' place in national distribution of income

A growing body of literature explores how individuals perceive inequality, with a particular emphasis on how they conceptualize their own socio-economic standing, view social structures and broader inequality trends within their societies. Recent studies employ advanced methodologies, often survey-based, to capture these perceptions. The first stream in literature explores perceptions of national income inequality or national social structure, identifying the extent to which individuals recognize the disparities between social classes in their societies (Niehues, 2014; Hauser and Norton, 2017; Gimpelson and Treisman, 2018; Engelhardt and Wagener, 2018). The second stream in literature instead focuses on individuals' perceptions of their relative income rank within national income distribution and biases in how people locate themselves relative to others. To quantify individual rank perceptions researchers typically use tools such as income ladders or percentile scales, where respondents indicate their perceived rank on numeric continuum (Cruces et al., 2013; Engelhardt and Wagener, 2018; Bublitz, 2022; Fernández-Albertos and Kuo, 2018).

Regardless of the way of measurement and across diverse settings, studies consistently demonstrate significant biases in how individuals perceive their own position in the income distribution.^1^ For example, Feichtmayer and Gründler (2021) report that approximately 40% of individuals in the OECD countries and around 37% worldwide believe that they are in the middle of the income distribution, even if objective measures contradict this perception. Feichtmayer and Gründler also find that among households in the lowest 30% of income distribution, 70% of households overestimate their relative standing. Similarly, Hoy and Mager (2021), in their comparative study of ten different countries, find that substantial proportions of respondents from the bottom two quintiles perceive themselves to be in the middle of the distribution, ranging from 37% in the United Kingdom to 63% in India Hoy and Mager, (2021), p. 316). Research from Sweden provides further insights into these patterns. Karadja et al. (2017) report that 68% of respondents underestimate their relative income position by more than 10 percentage points, in contrast to 6% who overestimate it. Results become more explicit when applying stricter criteria for accurate perceivers as those who correctly identify their income decile: 85.8% of respondents underestimate their position, while just 12.5% overestimate it. Also, findings of Cruces et al. (2013), analyzing data from Argentina, reveal that the most people misperceive their position in income distribution. They observe that 30% of respondents think that they are richer than they are and 55% think that they are poorer than in reality. Similarly, Fernández-Albertos and Kuo (2018), using data from Spain, report high level of misperceptions: Approximately 85% of respondents didn't manage to identify their place in income distribution correctly. Among them, over-estimators and under-estimators are nearly evenly divided, accounting for 40 and 45%, respectively. These findings together highlight the systematic nature of income rank misperceptions. Across diverse nations and methodological approaches, a clear majority of individuals, often exceeding tree-quarters, misperceive their income rank in one way or another.

Therefore, most researchers find the same pattern across different contexts when describing biases in perceptions of individual positions in income distribution. There is a center tendency of misperceptions Sattler-Bublitz et al., (2024), p. 29) or median bias (Hoy and Mager, 2021) when both rich and poor individuals view themselves as middle class. This aligns with frameworks of the “self-centered density function” (Knell and Stix, 2020, p. 2) or the “center bias” (Hvidberg et al., 2023, p. 3085). These studies reveal a positive correlation between perceived and objective income ranks; however, the most pronounced tendency is for individuals in the upper segment of the income distribution to underestimate their rank, whereas those in the lower segment often overestimate it.

Empirical evidence consistently show that erroneous beliefs are heterogeneous across population. For instance, Karadja et al. (2017) find that in Sweden several key sociodemographic characteristics improve the accuracy of relative income rank estimates, including years of education, cognitive abilities, self-reported media consumption and recent experience of upward mobility. However, the authors observe no significant correlation between the accuracy of perceptions and political orientation or the type of settlement (urban vs. rural). Further, adopting a broader perspective, Feichtmayer and Gründler (2021) examine misperceptions of social status across 97 countries and for 241,757 households. They find that older individuals, those with lower level of education and at the bottom of income distribution, have the tendency to overestimate their social status. In contrast to findings for Sweden, they find that being of left orientation is associated with less overestimation of social status. Busemeyer et al. (2024) explore the role of political attitudes and ideology for perceptions about individual relative income ranks using the data of the Konstanz Inequality Barometer (2020 and 2022). They argue that individuals tend to rely on their broader political beliefs when reflecting on their own position in income distribution and indeed find that in Germany politically conservative individuals underestimate their income rank, while those conservative in terms of economic ideology are more likely to overestimate it. Arin et al. (2024) conduct a comprehensive analysis of inequality perceptions in the United Kingdom and Germany, examining different dimensions: immigration, income inequality, and poverty. Their study reveals consistent overestimation of both poverty levels and share of those on top of the income distribution. They also find that gender, education, income, and political orientation are the significant determinants of misperceptions. For instance, women tended to overestimate the share of those under the poverty line, while men overestimate the income share of the richest. Although highly educated respondents showed more accurate perceptions of poverty levels, they are less precise about perceptions of income shares of those on top or bottom of income distribution.

In addition to individual-level determinants of misperceptions, contextual factors related to regional or cross-country differences such as regional- and country-level inequality and poverty rates also relate to income rank perceptions. Regional disparities in income distribution may amplify or mitigate biases, as individuals adjust their perceptions to localized norms and observable inequalities. Country differences also matter. Feichtmayer and Gründler (2021) document substantial cross-national differences in misperceptions. Interestingly, the respondents in higher developed countries such as Switzerland, the US, and Germany tend in general to overestimate their relative social standing, whereas individuals in Eastern Europe underestimate their own position. Arin et al. (2024) also find differences between the United Kingdom and Germany. The differences in all these instances could be due to various factors inherent to the countries such as history, economic growth and development, and the different institutions Gründler and Köllner, (2017).

### 2.2 Individuals' place in global distribution of income

The rise of globalization and inter-connectedness between countries, and advances in quality and availability of national-income data have made it necessary and possible to locate individuals on the global income scale. The integrated datasets containing the income data from most of countries worldwide now represent comprehensive global income database (Milanovic, 2016). These datasets allow for both cross-country comparisons and analyses of differences among residents of various nations. Thanks to those, objective income distribution worldwide and income inequality are increasingly being studied both within and across countries (e.g., Piketty, 2014; Hickel, 2017; Kanbur et al., 2022; Chancel and Piketty, 2021; Darvas, 2019; Milanovic, 2016).

The literature distinguishes between three main concepts of global inequality: unweighted international inequality or between country inequality, population-weighted international inequality, and global interpersonal inequality (Milanovic, 2006, 2013). Unweighted international inequality measures differences in per capita income across countries, calculated using GDP or average national income from household surveys without considering population size. Population-weighted international inequality incorporates also population sizes, providing an additional measure of inequality between countries. Global interpersonal inequality examines individual incomes worldwide by treating each person equally regardless of national affiliation. This latter approach conceptualizes the world as composed of individuals rather than nations and is of direct interest for the study of individual rank.

The global perspective is important as the place of individuals within national distribution and global distribution does not necessarily match. The individual position on global income rank is a result of both the individual position on national scale and the relative position of a country in a global context. Individuals from richer countries are naturally placed in the upper parts of the global distribution. However, over time, globalization has brought about the most profound redistribution and convergence of income ranks between the West, China, and other developing nations since the Industrial Revolution (Milanovic, 2024). Thus, the position of citizens of developed countries on the global scale has deteriorated over time, whereas the position of citizens of developing countries has improved (Milanovic, 2016).

Recent global processes and changes of global income distribution are often unknown to ordinary citizens across many countries regardless of the level of their development, making it difficult for ordinary people to understand their position globally. Indeed, the focus on the (perceived) interpersonal global income distribution results from the interest to explore whether people are aware of their position within the global income distribution. However, despite growing interest in measuring individual position in global income distribution, only few recent studies address individual perceptions of relative position in the global income distribution. Moreover, the analysis of (mis)perceptions of individual ranks in the global income distribution, defined as the discrepancy between perceived and actual income ranks is particularly rare in the literature (Fehr et al., 2022; Nair, 2018; Fehr et al., 2024). The examples of a global focus is research conducted by Fehr et al. (2022) in Germany and Nair (2018) in the United States, who examined how individuals form perceptions of their ranks in national and global income distributions, and how those perceptions causally relate to their national and global policy preferences. They find that almost half of the respondents underestimated their position in the global distribution. Similar to the studies on the national context Knell and Stix, (2020); Bublitz, (2022); Cruces et al., (2013), individuals tend to follow the scenario of center tendency (Sattler-Bublitz et al., 2024) or “middle-class” bias (Fehr et al., 2022). At the bottom of global income distributions individuals often overestimate their position in the global ranking, whereas those on the top tend to underestimate their position. Similarly, Sattler-Bublitz et al. (2024) explore how individuals in four European countries perceive their own income rank within the EU income distribution and observe the same tendency to lean toward the center. In contrast, some studies explore (mis)perceptions of between-country income inequality. For instance, Ziano et al. (2024) analyse how people estimate various indicators of economic development. Their findings suggest that Westerners tend to overestimate the wealth of developing and middle-income countries, underestimating the extent of global inequality. Therefore, despite the relatively limited number of studies in this area, all the previous studies about developed countries consistently reveal that individuals frequently misjudge their position within the global or supranational income distribution by mostly underestimating it together with the level of development and wealth of their own countries.

In addition to description of (mis)perceptions in global context, it is essential to understand the correlates of these (mis)perceptions. Yet, the study of individual correlates of perceptions of global inequality are limited, and most research simply argues that all individuals irrespective of their characteristics develop flawed perceptions of global income due to their reliance on their co-residents as their reference points. Nair (2018) claims that in advanced economies like the United States, reference groups are often limited to domestic peers, whose incomes tend to be higher at each percentile compared to the global distribution. As a result, individuals in such contexts systematically underestimate their global income rank while overestimating the global median income. Generally, misperceptions about income distribution are often more pronounced at the global level than in a national context, because individuals are more exposed to the information about domestic income than to the information about the global income. At the national level, people can make more accurate estimates about income distribution by observing their co-workers, neighbors, and social circles Nair, (2018), p. 5). Additionally, the media, political debates, and public reports often reinforce awareness about national income situation (Nair, 2018). By contrast, global income distribution receives far less attention in media and political discourse, particularly in developed countries where discussions of poverty in developing nations are limited. This imbalance further exacerbates biases when individuals assess their positions within global contexts (Nair, 2018).

## 3 Methodology

### 3.1 Survey data

To address our initial research questions, we utilize novel large-scale data collected through an online cross-sectional survey conducted in Italy in the months of November and December 2024 and in February and March 2025 (Kulic et al., 2025). The survey targeted individuals aged 18–70 and employing quotas to ensure representation of the Italian population across key socio-demographic variables, including gender, age, employment status, education level, and macro-region (North, Center, and South and Islands). The survey achieved a total sample size of 12,000 respondents, providing a robust foundation for in-depth analysis of income (mis)perceptions and their correlates across individual and contextual dimensions. The total sample after excluding cases with missing data on our core variables consists of 11,975 observations.

The research relies on questions assessing participants' perceived positions in national and global income distributions, socio-economic and demographic information, political orientation as well as the place of residence that was used as an indicator of geographical context.

### 3.2 Dependent variables

This study uses two dependent variables capturing misperceptions of individual income ranking: one at the national level and the other one at global level. *Misperceptions of individual position in the income distribution*, either national or global, was derived from difference between respondent's subjective and objective income ranks expressed on a 0–100 percentile scale. Therefore, positive values indicate overestimation of one's income rank, while negative values indicate underestimation. Larger absolute values indicate greater misperception in one direction or another. The variable is continuous and ranges from −100 to 100.

As follows, the first dependent variable, *Misperceptions of individual position in the national income distribution*, is derived from the following continuous measures:

- *Perceived income rank in national income distribution (in percentiles)*. Respondents' subjective income position was assessed by asking them to estimate the percentage of people in Italy with a lower net monthly per capita income than their own, thereby positioning themselves on a national income distribution scale ranging from 0 to 100%.
- *Objective income rank within national income distribution (in percentiles)*. Objective positioning was derived from reported household incomes. Respondents answered two questions addressing measurement of income. First, participants selected the income range corresponding to their actual household income (from *e*0–*e*250 up to “over *e*6,000”). Participants were also given the option to specify their exact household income, should they wish to do so. When no precise figure was provided, household income was estimated based on the previously chosen range, using the midpoint of the interval as a reference. Objective individual income rank at the national level was then calculated as self-reported household net average monthly income divided by the number of adult household members. This adjustment was essential for ensuring comparability across national and global scales, as the latter relies on the World Inequality Database, which considers adults as the primary units in household income shares. Respondents were ranked based on their net individual incomes, from the 1st to the 100th percentile. This ensures that each person's income rank reflects their relative position in the national distribution, making it directly comparable to subjective assessments of income rank.

In a similar fashion, the second dependent variable *Misperceptions of individual position in the global income distribution* is a difference between:

- *Perceived income rank in global income distribution (in percentiles)*. Respondents' subjective income position was assessed by asking them to estimate the percentage of world citizens with a lower net monthly per capita income than their own, thereby positioning themselves on a global income distribution scale ranging from 0 to 100%.
- *Objective income rank within the global distribution (in percentiles)*. To determine respondents' objective income rank within the global distribution, self-reported monthly household income data adjusted for the number of adult members in the household, and expressed in purchasing power equivalents (PPP, 2023 conversion rate) were matched with the World Inequality Database (WID).^2^ This database provides information on the historical evolution of the global distribution of income and wealth, both within and between countries, including pre- and post-tax income for individuals in different countries worldwide.^3^ Income cut-offs from this database showing global income thresholds for each percentile ranks in the global income distribution were compared and matched with the incomes from IneqPer data.^4^ For this study and within the WID, the indicator of post-tax income (“diinc”^5^.) for the world in 2023 was used, representing the sum of primary individual incomes across private and public sectors, excluding taxes but including in kind benefits. In the absence of available measures of disposable income without in kind benefits, this is the measure that most closely resembles net individual income from all sources that is collected within the IneqPer dataset. The WID population includes adults aged 20 and above, while the IneqPer sample includes those who are aged 18 and older, introducing minor inconsistencies. WID data use equal-split adult units, meaning that income is divided equally among all adult household members, and IneqPer income measures are harmonized accordingly. In summary, by comparing an individual's net income in Italy to global income distribution, we assign to individuals an objective percentile rank within the worldwide income distribution.

### 3.3 Independent variables

Following previous research, the study includes a range of key sociodemographic variables, political orientation and contextual variables as possible correlates associated with income rank misperceptions both at national and global levels. Regression models incorporate the following independent variables: gender, age, education, employment status, migration background, urban–rural residence, and left–right political orientation.

Gender is coded as a binary variable (men and women). Age is grouped into six categories to reflect different life stages and capture possible cohort effect (18–24, 25–34, 35–44, 45–54, 55–64, and 65–70). The level of education is classified into three levels: Low-Up to elementary school, Medium-High school, and High-level-Bachelor degree and above. Employment status differentiates between employed, unemployed, inactive, and other. Migration background reflects the respondents' and their parents' country of birth and citizenship status. Respondents are classified into three groups: no migration background (if both the respondent and their parents are Italian), first generation (if respondent was born abroad), and second generation (if respondent was born in Italy but at least one of the parents is an immigrant). The type of settlement distinguished between rural and urban areas.^6^ Political orientation derives from self-placement on the left-right scale ranging from 1 (“far left”) to 10 (“far right”) and recoded into four categories: left (1–3), center (4–7), right (8–10), and missing (no response).

### 3.4 Contextual variables

The analysis includes indicators for 20 Italian regions and four macro-areas: North-West, North-East, Center, and South and Islands. These contextual variables are analytically relevant due to Italy's persistent territorial disparities in economic development between less developed Southern regions and more developed regions from the North and the Center (D'Adamo et al., 2021, 2025).

## 4 Analytical strategy

The analysis is organized into two main steps: first, the distributions and descriptive analyses of subjective and objective ranks within both national and global income distributions, along with the corresponding (mis)perceptions, are presented. Second, given the continuous nature of the dependent variable representing (mis)perceptions, ordinary least squares (OLS) regression models are employed to examine the individual and contextual correlates of income rank misperceptions at both the national and global levels.

Two main model specifications were estimated: the first considers only individual-level correlates: gender, age, education, employment status, migration background, the type of settlement (urban or rural), and political orientation. The second specification extends the model by adding covariates for 20 regions. Finally, additional analyses examine the interaction effects between education and gender, as well as between education and age, and include separate models for the four Italian macro-areas, to explore whether basic associations vary across different population subgroups.

As diagnostic tests indicated heteroscedasticity and non-normality of residuals^7^, all models were estimated using robust standard errors to obtain consistent inference. Moreover, given the large sample size, both *t*-tests and *F*-tests retain their reliability even under deviations from normality. All analyses rely on weighted results to ensure representativeness. The weights adjust for gender, age, macro-area, employment and education variables to match the sample achieved proportions to predefined quotas.

## 5 Results

**Figure 1:**
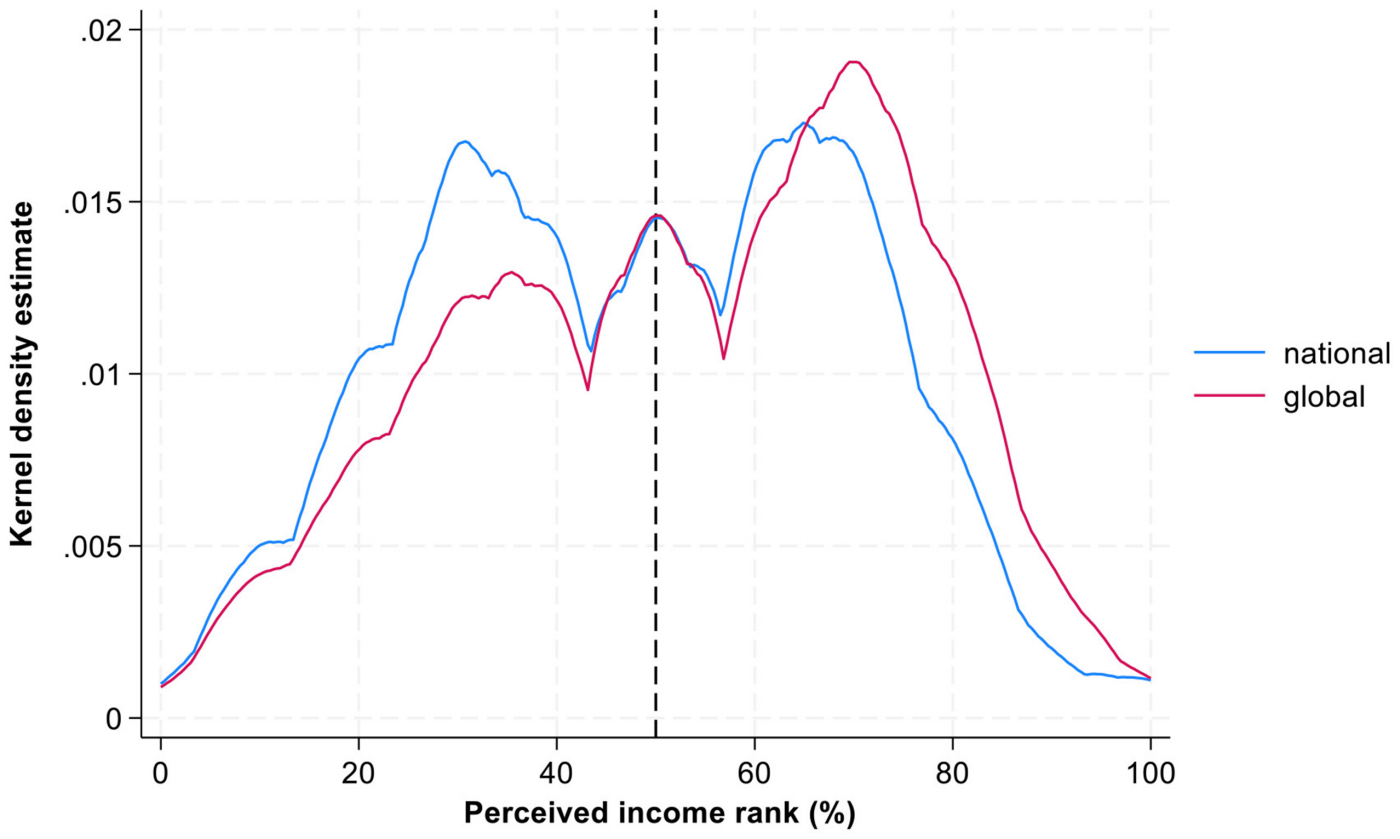
Perceived position in national and global income distribution: Kernel density estimation. Source: IneqPer 2024, Italy, *N* = 11,975.

Table 1 presents the descriptive statistics for the main variables in the study. These statistics provide a comprehensive overview of the sample characteristics for gender, age, education, employment status, migration background, type of settlement, political orientation, and regions as the key factors in understanding variations in income rank perceptions and misperceptions. The table also provides an overview of the mean subjective and objective income rank, and the resulting (mis)perceptions. Income is a key variable in the study. The average income, calculated on a per capita basis using the equal-split adult method, amounts to approximately *e*1,128.53, with a standard deviation of *e*2,602.30, indicating substantial variability in income across the sample. After adjusting for purchasing power parity (PPP), the mean income rises slightly to *e*1,252.03, reflecting lower relative costs of living in Italy and economic equivalencies. Table 1 is accompanied with Figure 1 illustrating the distribution of individuals' subjective perceptions of their income rank in national and global income distribution.

**Table 1:** Descriptive analysis of the sample (*N* = 11,975).

**Frequency**			
**Variable**		**Percent**	**Variable**	**Percent**
Gender			Macro-region
Man		48.81	North-West	27.29
			North-East	18.68
Woman		51.19	Center	19.86
			South and Islands	34.17
Age			Regions
18–24		10.16	Lombardy	17.03
25–34		15.82	Liguria	2.35
35–44		18.88	Piedmont	7.62
45–54		23.99	Aosta Valley	0.29
55–64		21.70	Veneto	7.52
65–70		9.44	Trentino-Alto Adige	1.04
			Emilia-Romagna	8.28
Education			Friuli Venezia Giulia	1.85
Low		33.43	Marche	2.61
Medium		42.86	Lazio	10.25
High		23.71	Tuscany	5.77
			Umbria	1.24
Migrant background			Abruzzo	1.81
No migrant background		90.07	Molise	0.49
1st generation		4.43	Campania	8.33
2nd generation		5.49	Apulia	7.72
			Basilicata	0.94
Employment status			Calabria	2.91
Employed		57.99	Sardinia	3.59
Unemployed		8.42	Sicily	8.38
Inactive		30.44	
Other		3.16	
Political orientation			Self-definition of residence
Left		20.85	Urban	92.28
Center		41.98	Rural	7.72
Right		21.90	
Missing		15.27	
	**Mean and standard deviation**			
**Variables**	**Mean**	**SD**	**Min**	**Max**
Income (calculated as equal-split adults)	1,128.54	2,602.31	0.167	160,000
Income (calculated as equal-split adults, adjusted per ppp)	1,249.73	2,881.58	0.185	177,000
Subjective income rank in national income distribution	48.36	21.65	0	100
Objective income rank in national income distribution	50.22	28.89	0	100
(Mis)perception of income rank in national income distribution	−1.86	33.59	−98	99
Subjective global income position	53.54	22.30	1	100
Objective global income position	56.68	20.64	1	100
(Mis)perception on global income distribution	−3.14	28.05	−89	99

Figure 1 visualizes the perceived income ranks of Italians in both national and the global income distributions using kernel density estimations. The data reveal moderate-positive correlation between perceived income ranks at both national and global levels (*r* = 0.57, *p* < 0.05), showing that perceptions go in the same direction. At the same time, some differences between two distributions are notable. Within the national income distribution, perceptions exhibit a clear bimodal structure with the peaks around the 40th and 70th percentiles. This bimodality suggests the presence of two clusters in Italians' perceptions: one group aligns itself with the lower-middle class, while another identifies as upper-middle class. By contrast, perceptions of income rank within the global income distribution presents a unimodal pattern, with a dominant peak around the 70th percentile. This suggests that Italians tend to place themselves more often in the upper part of global income hierarchy.

### 5.1 Perceived individual income ranks and objective position in national income distribution

**Figure 2:**
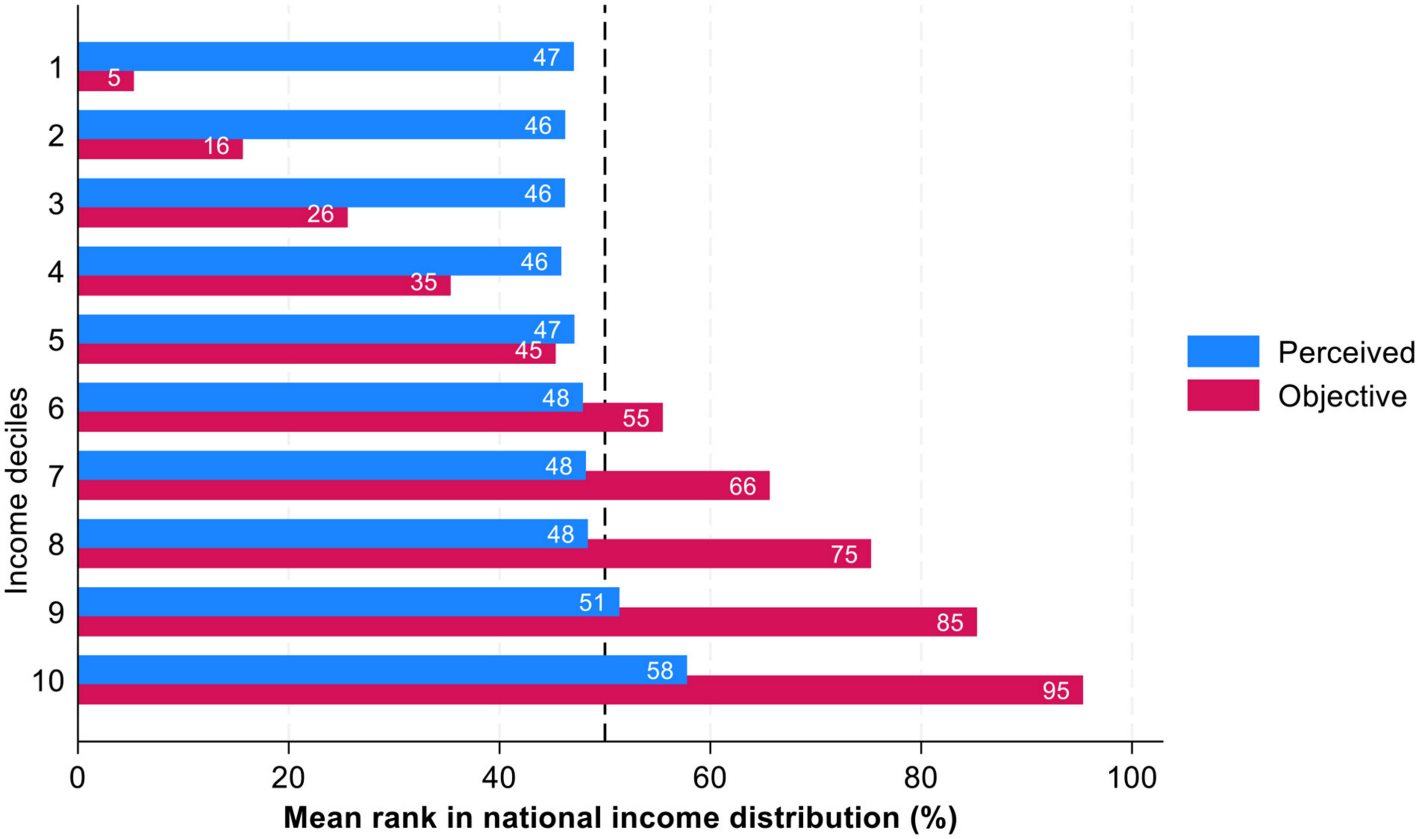
Perceived and objective position in income distribution across income deciles: average ranks in percentages. Source: IneqPer 2024, Italy, *N* = 11,975.

This section examines the relationship between individuals' perceived positions in the national income distribution and their objective positions, with a focus on systematic patters of misperceptions across income groups. Figure 2 categorizes respondents by income deciles and compares their subjective and objective income ranks within the Italian income distribution.

The divergence between objective and subjective positions highlights the systematic misperceptions across income groups. There is a strong tendency for individuals to regress toward the mean, with those in lower income deciles overestimating their relative position and those in higher deciles underestimating it. For instance, the average perceived percentile in the 1st income decile is 46, whereas in the 10th income decile, it is 57. Despite the significant disparity in actual income levels between these deciles, the perceived difference amounts to just 11 percentage points. This pattern underscores a pronounced bias: the further individuals are from the middle of the income distribution, the more pronounced their misperception of their position. In contrast, individuals in the middle of the distribution exhibit the smallest gap between their objective and subjective positions, indicating a closer alignment between perception and reality.

This pattern reflects and confirms for Italy the well-documented phenomenon of the bias to the median or center bias where individuals across income groups tend to cluster their self-assessments around the middle of the income distribution. Such findings align with previously mentioned literature, which documents “center bias” (Hvidberg et al., 2023), “median bias” (Hoy and Mager, 2021), and “middle-class bias” (Evans and Kelley, 2004; Fehr et al., 2022).

Table 2 provides further clarifications and reveals insights into income rank misperceptions defined as difference between perceived and actual income rank in national distribution (see also Supplementary Figure A1). Interestingly, a majority of respondents (53%) with different degree underestimate their position in the income distribution, believing they are ranked lower than they actually are. In contrast, 46% overestimate their income rank, positioning themselves higher than their objective place. A small fraction, 1%, or 141 individuals out of 11,975, managed to exactly align their perceived and actual ranks (Table 2). These results confirm the previous findings for Spain and Argentina where both groups of over- and under-estimators are well populated (Cruces et al., 2013; Fernández-Albertos and Kuo, 2018), but not for Sweden where the majority underestimate their income rank in national income distribution (Karadja et al., 2017).

**Table 2:** Misperceptions in the national income distribution: the underestimators, overestimators and accurate perceivers.

**Misperceptions of income position at national level**	**Freq**.	**Percent**
Under-estimators	6,372	53.22
Accurate perceivers	141	1.17
Over-estimators	5,462	45.61
Total	11,975	100.00

The extent of deviation among a substantial portion of the population highlights the widespread nature of income rank misperceptions and raises questions about the factors driving these biases. These findings have critical implications for understanding public attitudes toward income inequality and the potential mismatch between subjective experiences and objective realities.

**Figure 3:**
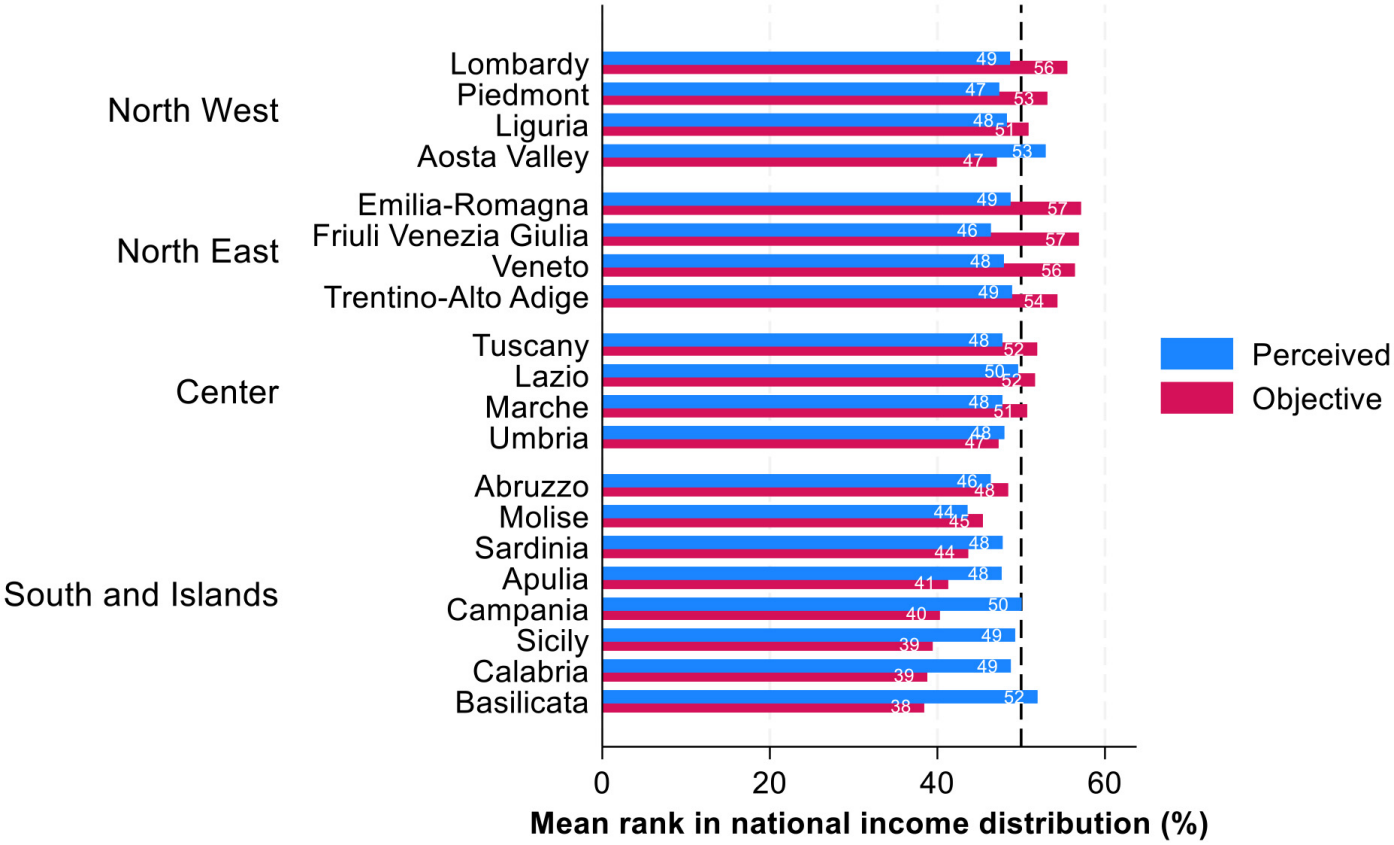
Perceived and objective position in income distribution across Italian regions: average ranks in percentages. Source: IneqPer 2024, Italy, *N* = 11,975.

Regional disparities also reveal systematic differences in income rank misperceptions (Figure 3). Here, we can see the same pattern and clear North–South divide: residents of more affluent Northern regions tend to underestimate their relative income rank in national distribution while residents of the poorer Southern regions overestimate their income ranks. For instance, in the economically prosperous regions of Lombardy, Veneto, and Emilia-Romagna, the objective income ranks are relatively high, on average 57–58th income percentiles, yet average perceived positions are between the 48th and 49th percentiles. Conversely, in less affluent regions such as Calabria, Basilicata, and Sicily, where average objective income ranks are 39 percentiles, individuals' perceived income positions surpass their objective ranks by over 10 percentile points. In Calabria and Sicily, the average perceived income rank is 49, and in Basilicata 52. In other words, in less prosperous regions there is an overestimation of own position, in comparison to prosperous regions where the underestimation is visible.

As for general tendency across income deciles, for Italian regions we can observe a central tendency bias: individuals across regions tend to perceive themselves closer to the middle of the income distribution regardless of average income ranks in their regions.

### 5.2 Perceived individual income ranks and objective position in global income distribution

**Figure 4:**
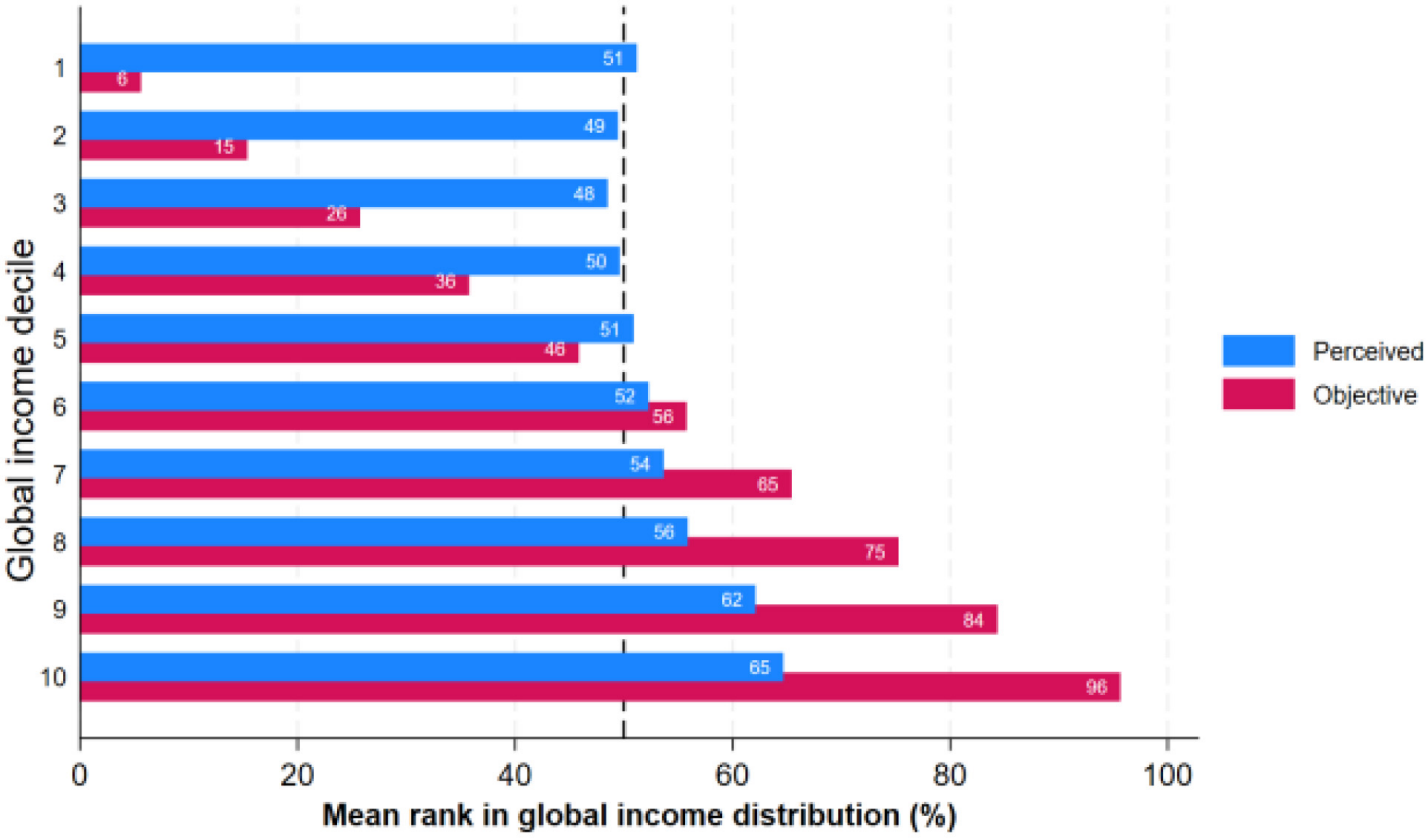
Perceived and objective position in global income distribution across global income deciles: average ranks in percentages. Source: IneqPer 2024, Italy, *N* = 11,975.

In the same way as for national income distribution, respondents tend to place themselves near the middle of the global income distribution, regardless of their actual income levels (Figure 4). Similar to the national income distribution, this pattern reflects a central tendency bias, where individuals gravitate toward the median. Similar to the findings above, this trend is more pronounced for the extreme deciles: individuals in the lowest global income deciles tend to overestimate their position, while those in the highest deciles underestimate it.

For example, those Italians who are in the 1st global income decile perceive themselves to be at the 51st percentile globally, while in reality, they are on average positioned around the 6th percentile. Conversely, individuals in the top 10th global decile (the richest) believe that they are at the 65th percentile, though their actual position is closer to the 96th percentile. Respondents that are in the 5th and 6th global deciles are the only groups that come close to accurately identifying their global standing: they perceive themselves to be at the 51st and 52nd percentiles, while their objective positions are around the 46th and 56th percentiles, respectively. The finding aligns with results from previous studies in Germany (Fehr et al., 2022) and the United States (Nair, 2018), which also identified a middle-class bias in global income perceptions.

The distribution of misperceptions of global income ranks shows almost the same pattern as for misperceptions of income ranks in national income distribution. About 54% of respondents underestimate their global income rank, 44% overestimate it, and 1.5% are accurate in their estimates (Table 3, Supplementary Figure A2).

**Table 3:** Misperceptions on the global income distribution: the underestimators, overestimators and accurate perceivers.

**Misperceptions of income position at global level**	**Freq**.	**Percent**
Under-estimators	6,493	54.22
Accurate perceivers	181	1.51
Over-estimators	5,301	44.27
Total	11,975	100.00

**Figure 5:**
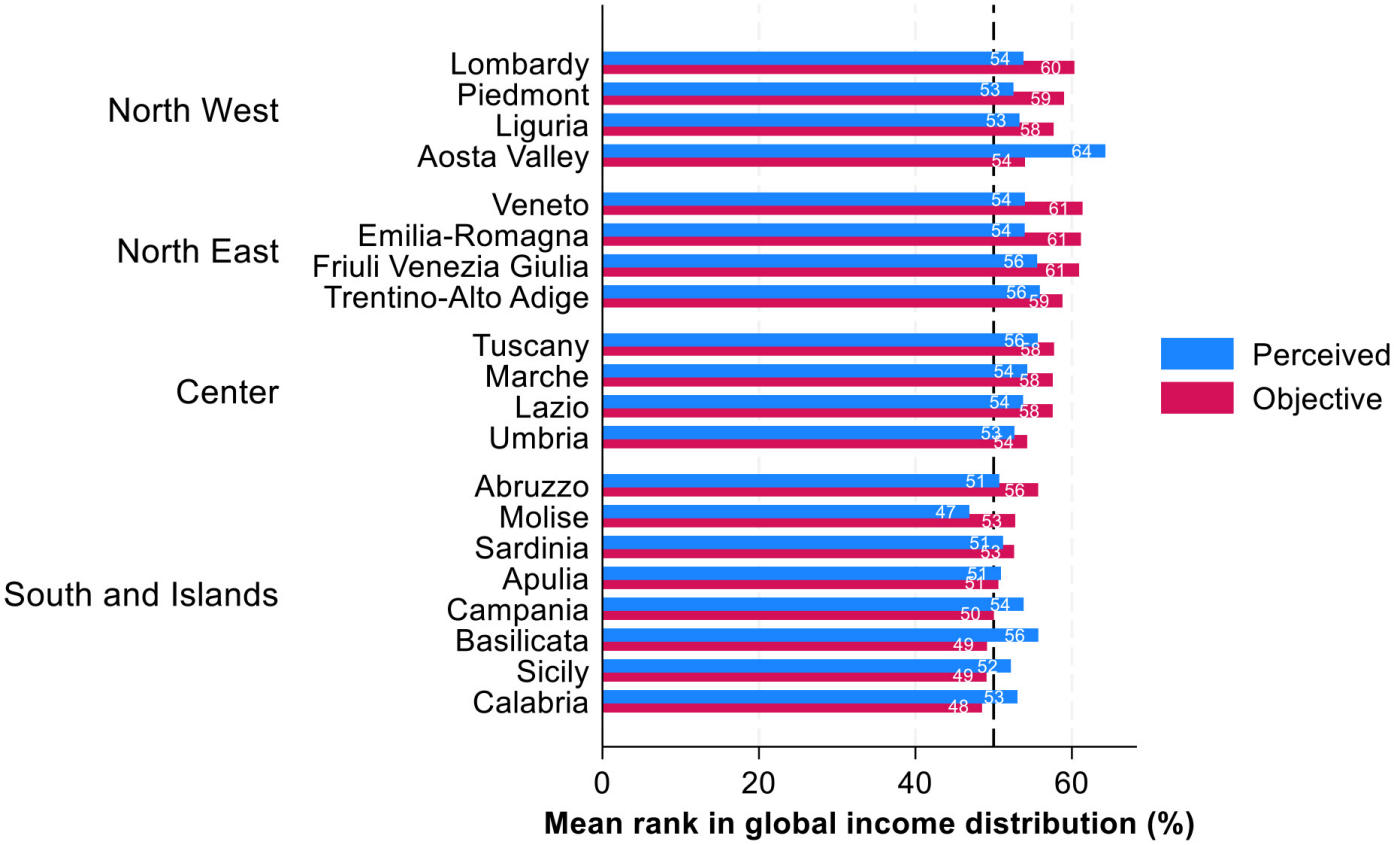
Perceived and objective position in global income distribution across Italian regions: average ranks in percentages. Source: IneqPer 2024, Italy, *N* = 11,975.

Center tendency bias for regions can also be observed for perceptions of individual ranks on a global scale. Similar to the findings about perceptions of individual ranks within national context, Figure 5 displays the perceived and objective income ranks of respondents in global income distribution by their regions of residence. In line with center tendency rule, individuals living in more affluent Northern and Central regions generally tend to underestimate their global objective position, while those residing in poor Southern regions tend to overestimate their global objective position. The only exception in the Northern regions is Aosta Valley, while in the Southern regions, the exceptions are Abruzzo and Molise. For instance, in the more developed regions such Lombardy, the mean objective rank in global income distribution is the 60th percentile whereas the perceived global income rank is at 54th percentile. Similarly, in Piedmont, the objective mean for global rank is 59th percentile in comparison to subjective mean global rank of 53rd percentile; this applies also to Liguria where the objective rank is 58th percentile vs. the subjective rank of 53rd percentile. On the contrary, in the Southern region of Sicily, the mean objective income rank is 49th percentile in comparison to the subjective income rank of 52nd percentile. In Calabria, another Southern region of Italy, the mean objective global rank is 49th percentile in comparison to 53rd percentile as subjective rank. This confirms the general finding that those who are poorer either as individuals or living in poorer regions tend to overestimate their position toward the mean, whereas those who are richer individually and living in richer regions, tend to underestimate their position toward the mean.

### 5.3 Heterogeneity of misperceptions of income ranks in national and global income distribution

**Figure 6:**
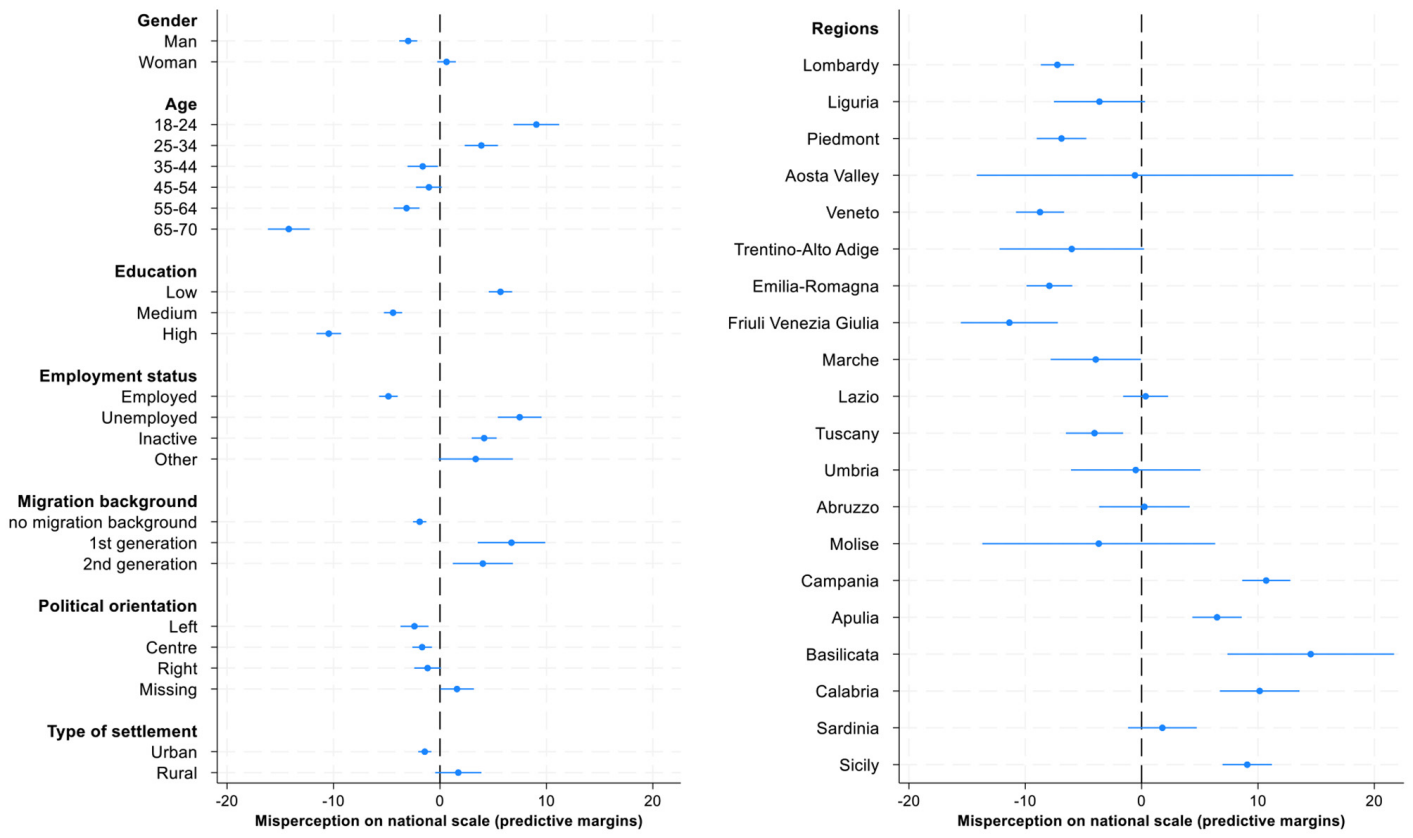
Predictive margins of individual and contextual correlates of income ranks misperception in national income distribution, 95% CI. Source: IneqPer 2024, Italy, *N* = 11,975.

**Figure 7:**
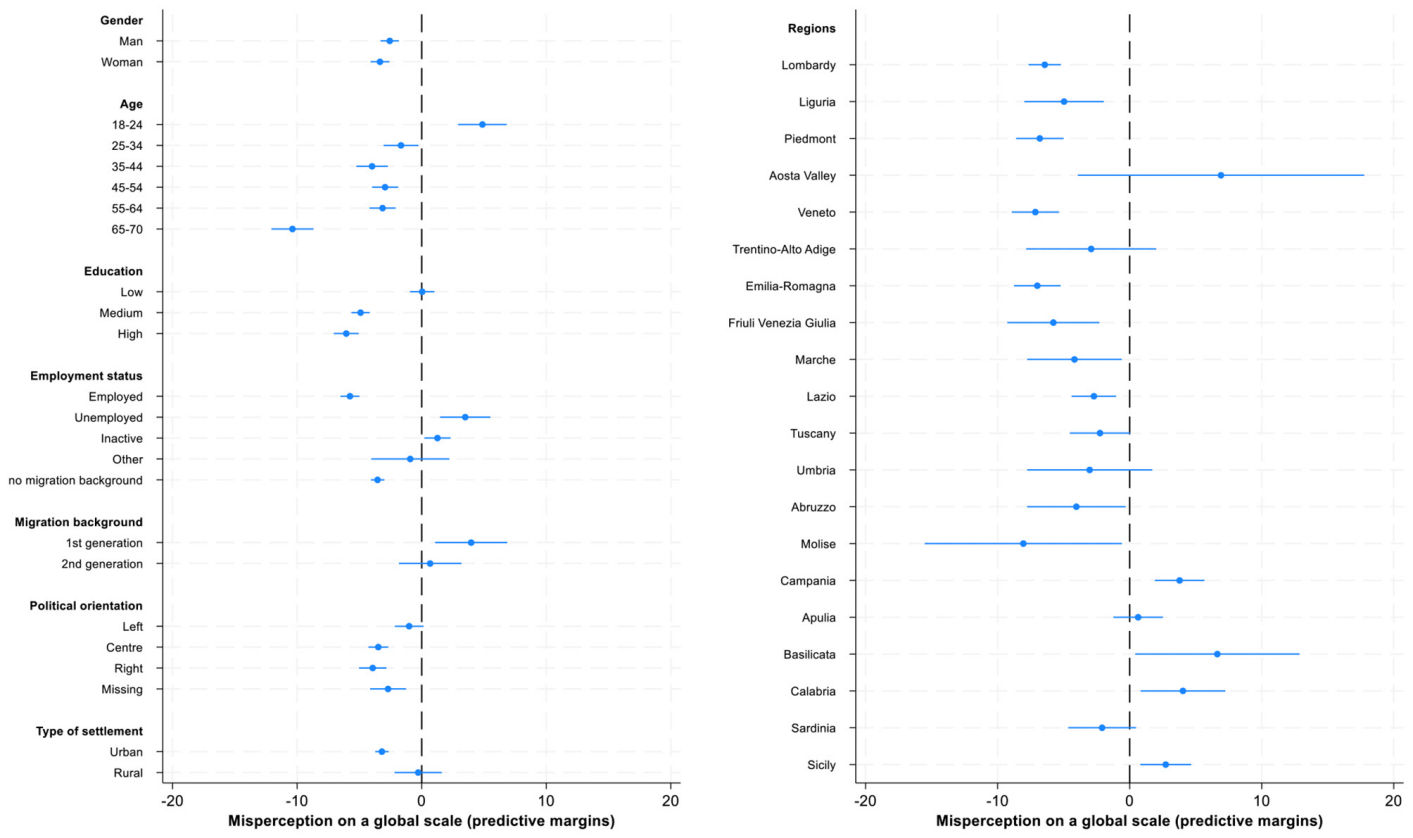
Predictive margins of individual and contextual correlates of income ranks misperception in global income distribution, 95% CI. Source: IneqPer 2024, Italy, *N* = 11,975.

This section investigates which social groups are more prone to misperceive their income ranks both in national and global context, and explores how these misperceptions vary across sociodemographic and contextual divides. The current analysis consists of a series of regression models to examine the correlates of income rank misperceptions within country and within world in greater detail. Since our dependent variables, misperceptions of income rank, either in national or global income distribution, can take both positive and negative values, the interpretation of raw regression coefficients is not always intuitive. While the coefficients show the direction and relative magnitude of group differences compared to the reference category, they don't reveal whether a given group's estimates values are closer to zero (that means more accurate perceptions) or further from it (indicating larger misperceptions). To enhance interpretability, the regression coefficients (reported in Table 4) are complemented with predictive margins (Figures 6, 7, Supplementary Tables A1, A2) that translate estimated results into predicted values of misperceptions for each group, holding other variables constant. This approach allows us to directly observe and compare the degree of misperception across multiple categories.

**Table 4:** OLS Regressions of misperception of income position in national and global income distribution (*N* = 11,975), 95% CI.

**Dependent variable**	**Misperception of income position in national income distribution**	**Misperception of income position in global income distribution**
Woman (Ref: Man)	3.60^***^	−0.78
	(0.636)	(0.552)
25–34 (Ref: 18–24)	−5.17^***^	−6.54^***^
	(1.353)	(1.231)
35–44 (Ref: 18–24)	−10.67^***^	−8.86^***^
	(1.343)	(1.216)
45–54 (Ref: 18–24)	−10.09^***^	−7.82^***^
	(1.285)	(1.163)
55–64 (Ref: 18–24)	−12.19^***^	−8.02^***^
	(1.265)	(1.142)
65–70 (Ref: 18–24)	−23.25^***^	−15.25^***^
	(1.409)	(1.250)
Medium education (Ref: low education)	−10.09^***^	−4.95^***^
	(0.728)	(0.640)
High education (Ref: low education)	−16.13^***^	−6.10^***^
	(0.858)	(0.752)
Unemployed (Ref: employed)	12.33^***^	9.25^***^
	(1.167)	(1.124)
Inactive (Ref: employed)	8.99^***^	7.03^***^
	(0.816)	(0.726)
Other (Ref: employed)	8.20^***^	4.84^***^
	(1.840)	(1.653)
1st generation (Ref: no migrant background)	8.62^***^	7.51^***^
	(1.658)	(1.512)
2nd generation (Ref: no migrant background)	5.93^***^	4.22^***^
	(1.486)	(1.321)
Center (Ref: left)	0.72	−2.48^***^
	(0.808)	(0.706)
Right (Ref: left)	1.23	−2.92^***^
	(0.924)	(0.808)
Missing (Ref: left)	3.99^***^	−1.69^*^
	(1.071)	(0.964)
Rural (Ref: urban)	3.15^***^	2.91^***^
	(1.157)	(1.012)
Liguria (Ref: Lombardy)	3.61^*^	1.46
	(2.130)	(1.658)
Piedmont (Ref: Lombardy)	0.35	−0.37
	(1.314)	(1.109)
Aosta Valley (Ref: Lombardy)	6.67	13.35^**^
	(6.978)	(5.569)
Veneto (Ref: Lombardy)	−1.49	−0.71
	(1.285)	(1.107)
Trentino-Alto Adige (Ref: Lombardy)	1.23	3.52
	(3.258)	(2.591)
Emilia-Romagna (Ref: Lombardy)	−0.69	−0.57
	(1.242)	(1.097)
Friuli Venezia Giulia (Ref: Lombardy)	−4.13^*^	0.65
	(2.251)	(1.884)
Marche (Ref: Lombardy)	3.30	2.25
	(2.111)	(1.935)
Lazio (Ref: Lombardy)	7.58^***^	3.72^***^
	(1.224)	(1.063)
Tuscany (Ref: Lombardy)	3.19^**^	4.18^***^
	(1.457)	(1.328)
Umbria (Ref: Lombardy)	6.73^**^	3.40
	(2.935)	(2.497)
Abruzzo (Ref: Lombardy)	7.48^***^	2.39
	(2.114)	(2.002)
Molise (Ref: Lombardy)	3.56	−1.62
	(5.158)	(3.860)
Campania (Ref: Lombardy)	17.95^***^	10.21^***^
	(1.285)	(1.146)
Apulia (Ref: Lombardy)	13.72^***^	7.07^***^
	(1.307)	(1.146)
Basilicata (Ref: Lombardy)	21.78^***^	13.07^***^
	(3.725)	(3.233)
Calabria (Ref: Lombardy)	17.39^***^	10.46^***^
	(1.893)	(1.754)
Sardinia (Ref: Lombardy)	9.02^***^	4.34^***^
	(1.676)	(1.453)
Sicily (Ref: Lombardy)	16.32^***^	9.16^***^
	(1.307)	(1.166)
Constant	2.16	3.13^**^
	(1.658)	(1.493)
**Observations**		
*R*-squared	11,975	11,975

Standard errors in parentheses.

^***^p < 0.01, ^**^p < 0.05, ^*^p < 0.1.

Table 4 shows how demographic, socioeconomic characteristics, and political orientation along with geographical characteristics correlate with misperception of income ranks in national and global income distributions. The table indicates significant differences in misperceptions across groups. Figure 6 translates the coefficients in predictive margins for national income distribution. The *x*-axis represents the percentage point deviation in income rank perception with zero indicating accurate perception, while the *y*-axis lists various individual and contextual characteristics. Given that the dependent variable ranges from −100 to 100%, the estimates can be interpreted directly in terms of percentage point difference: positive values indicate an overestimation relative to the objective position, whereas negative values indicate an underestimation of objective income ranks.

The results show a statistically significant gender differences in misperceptions at national level: men are more likely to underestimate their position compared to women (*b* = 3.60, *p* < 0.01), who tend to accurately perceive their income rank. Age is important for misperceptions: The younger people are more optimistic about their income ranks. Young adults (18–24 years old) tend to overestimate their income rank by 9 percentage points. As people age, they become more realistic about their position in income distribution. Those who are 25–34 years old demonstrate less overestimation (4 percentage points) then members of the youngest group. Middle-aged people from 35 to 44 and 45 to 54 years old are rather accurate in the estimates of their own position in income distribution (−2 and −1 percentage points). In contrast, older cohorts tend to underestimate their income rank. This underestimation intensifies with age. Those who are from 55 to 64 years old think that their position is 3 percentage points lower than it is, and those who are over 65 think that they are 14 percentage points lower in income distribution compared to the real position.

Educational attainment is another strong correlate of misperceptions. Individuals with lower level of education overestimate their income rank by 5.68 percentage points, while those with medium (*b* = −10.06, *p* < 0.01; predictive margins is −4.40) and high levels of education (*b* = −16.13, *p* < 0.01; predictive margins is −10.44) show a tendency to underestimation. This pattern partially reflects previous findings about median bias of people located in lower- and upper-income deciles. Education in this context can be considered more as an indicator of a position in social hierarchy then as a source of unbiased information about individual positions in income distribution. This approach provides us with the reasoning why highly educated people underestimate their income rank by 10 percentage points. Differences also emerge by employment status: the unemployed significantly overestimate their rank by 7.49 percentage points, employed on the contrary generally underestimate their rank by 4.84 percentage points.

Regarding migration background, natives underestimate their position in income distribution, while immigrants, overestimate their position. Specifically, first-generation migrants show the highest levels of overestimation (7 percentage points), followed by second-generation migrants (4 percentage points), and natives slightly underrate their position by on average 2 percentage points. Overall, there is a clear pattern: low-resource individuals overestimate their position, while high-resource individuals underestimate their position in the income distribution. There is a difference in misperceptions between individuals living in urban and rural areas. Respondents in urban areas show slightly larger level of under-estimation of their income position (1.43 percentage points), in comparison to residents of rural areas that tend to be rather accurate.

Regional disparities are pronounced and align with broader economic divides in Italy. In this context, we can also identify evidence of the median bias: residents of more prosperous northern regions, for example Veneto, Friuli Venezia Giulia, and Lombardy, tend to underestimate their position in national income distribution, while residents of Southern regions, such as Campania, Basilicata, and Sicily, exhibit significant overestimation of their income rank. These findings are consistent with Italy's well-documented North–South divide in economic opportunities and income levels. The observed overestimation in the South may stem from a combination of lower exposure to national benchmarks and a greater reliance on local comparisons.

The following analysis contributes to the previous literature by systematic examination of the correlates of misperceptions of global income position. The same modeling strategy is applied as for misperceptions at the national level: misperceptions at global level are regressed on socio-demographic variables and 20 Italian regions (Table 4). Figure 7 presents the predictive margins with 95% confidence intervals derived from the OLS regression analyzing the misperception of income position in the global income distribution (Table 4). As before, misperception is defined here as difference between an individual's subjective and objective income percentile position in global income distribution; hence, lower values indicate greater underestimation, while higher values suggest overestimation.

The results show that both men and women tend to underestimate their position: the average underestimation is 3 percentage points yet with no significant difference across two groups. Younger adults, particularly those under 24, tend to overestimate their relative income position by 5 percentage points. In contrast, older age groups tend to underestimate their income rank on the global scale: older adults (65–70) show the most salient underestimation by 10 percentage points, whereas the other age groups are closer to accurate perceptions: they underestimate their position by 2-4 percentage points. Hence, the older the individuals are, the more they tend to underestimate their income percentile position on the global scale. Regarding education, highly and medium educated individuals tend to underestimate their position in global distribution (for 6 and 5 percentage points, respectively) while low educated show no bias in estimates. Unemployed respondents tend to overestimate their position by three points, while employed tend to underestimate their position by six points. Migration background also associates with individual misperceptions. First-generation migrants overestimate their income position by four points, while those without a migration background show a tendency to underestimate it by 3.5 percentage points. The second-generation migrants are relatively accurate in their estimates. Regarding political orientation, respondents having left political view perceive their income rank on global scale almost correctly (−1 percentage points), while those who positioned themselves at the center or right show a larger underestimation of between 3 and 4 percentage points. Those who preferred not to disclose their political orientation similarly underestimate their position, by approximately 2.7 percentage points. Respondents living in urban areas tend to underestimate their global income position by approximately 3 percentage points while those residing in rural contexts show no bias. Regarding the contextual correlates, residents of Northern and Central Italy (e.g., Lombardy, Emilia-Romagna, Tuscany) tend to underestimate their position, whereas individuals from Southern regions (e.g., Calabria, Sicily, Campania) tend to overestimate it. In Lombardy, Piedmont, and Liguria, as the three richest Italian regions (D'Adamo et al., 2021, 2025), the underestimation is approximately from 5 to 7 percentage points. Differently, in Southern regions there is an overestimation of global position, the largest of which in Basilicata (7 percentage points), followed by Calabria and Campagna (approximately at 4 percentage points). However, among the Southern regions, there are three exceptions: respondents from Sardinia, Abruzzo, and Molise underestimate their position by 2, 4, and 8 percentage points respectively. The results overall show significant similarity to previous findings about misperceptions of income ranks in national income distribution.

## 6 Further empirical considerations

**Figure 8:**
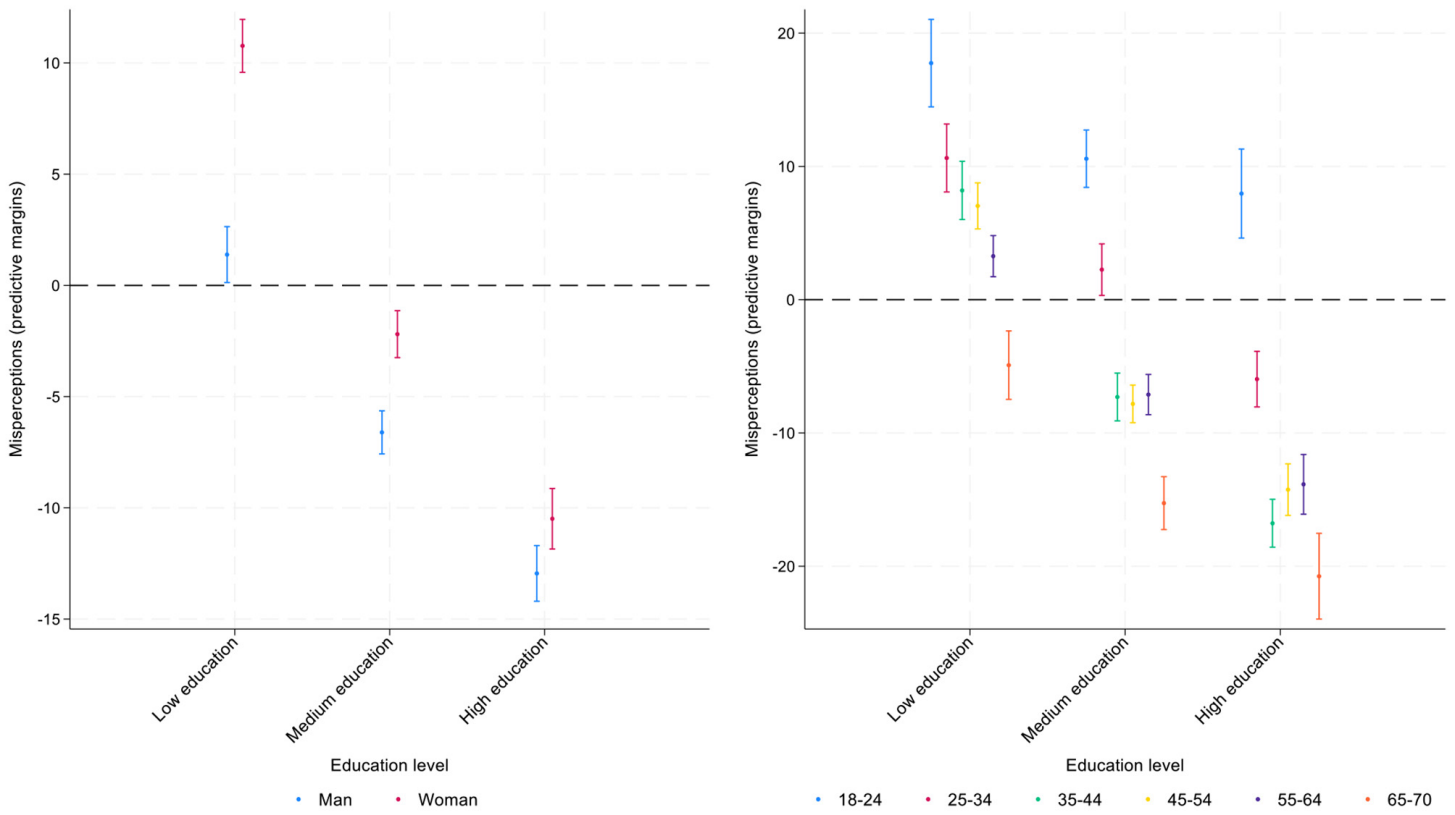
Interactions of education with gender **(panel a)** and age **(panel b)**. Predictive margins for misperceived income ranks in the national income distribution, 95% CI.

**Figure 9:**
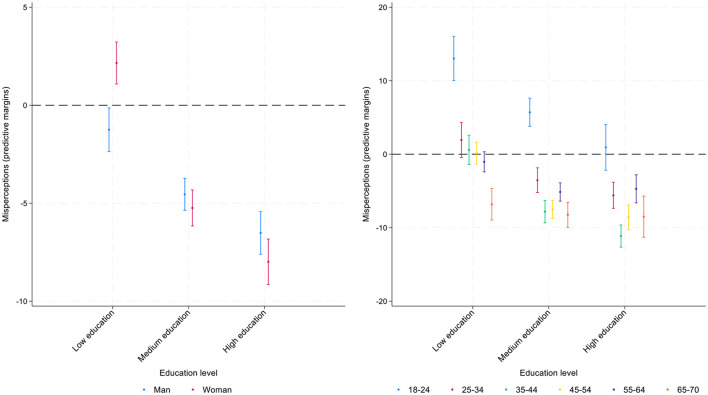
Interactions of education with gender **(panel a)** and age **(panel b)**. Predictive margins for misperceived income ranks in the global income distribution, 95% CI.

While main effects described above identify general group patterns, an intersection between different demographic characteristics provide a more nuanced picture on misperceptions. For instance, both age and gender may substantially moderate the main effects of other variables including individual education as one of the most important correlates of misperceptions (Karadja et al., 2017). Moreover, the direction and the magnitude of regression coefficients may vary across geographical contexts. Further analyses include the interaction terms for gender and education with age (Figures 8, 9, Supplementary Tables A3–A6) and regional variation across Italy's four geographical regions for all present variables (Figures 3, 4, Supplementary Tables A7, A8).

Regarding the interaction between education and gender (Figure 8a), in misperceiving national income distribution, the data show that women with low levels of education are more optimistic in their assessment of their relative income position compared to men with low levels of education: they overestimate their relative income rank, but men with low levels of education are almost accurate in their assessments. Overall, there is a substantial difference between gender gaps across the three groups of highly, medium, and low educated respondents: the gaps are largest for low educated and lowest for highly educated. However, both women and men among high and mid-educated underestimate their position in the income scale.

Age moderates the effects on education on misperceptions of one's position in the income distribution (Figure 8b). While people with low levels of education overestimate their position in the income distribution until fairly late in life (age 65), people with mid- and higher levels of education reduce their optimism earlier.

When examining the interaction between education and gender in evaluating the misperception of respondents' global income position (Figure 9a), the largest gender gap is found among respondents with low levels of education: women tend to show a slight overestimation, while men tend to show slight underestimation. Among the mid- to highly educated, both men and women underestimate their position similarly, with women slightly more than men (approximately −8 vs. −5 percentage points) among highly educated.

Regarding the interaction between education and age, among respondents with low levels of education, the youngest age group (18–24) is the one that overestimates their position the most (approximately by 13 percentage points), while the oldest group (65–70) underestimates the most (approximately by −9 percentage points). Overall, the gap between young and old respondents is highest among low educated and lowest among highly educated.

When comparing perceptions of global and national income ranks, the findings are similar regarding the moderating effects of gender. Some differences, however, emerge concerning the moderating role of age. Age consistently moderates the effect of education on income rank misperceptions in the national context across all age groups, but its influence is less pronounced for global rank misperceptions: significant interactions are observed only among older respondents with medium or high education, who tend to underestimate their position less than other age groups.

**Figure 10:**
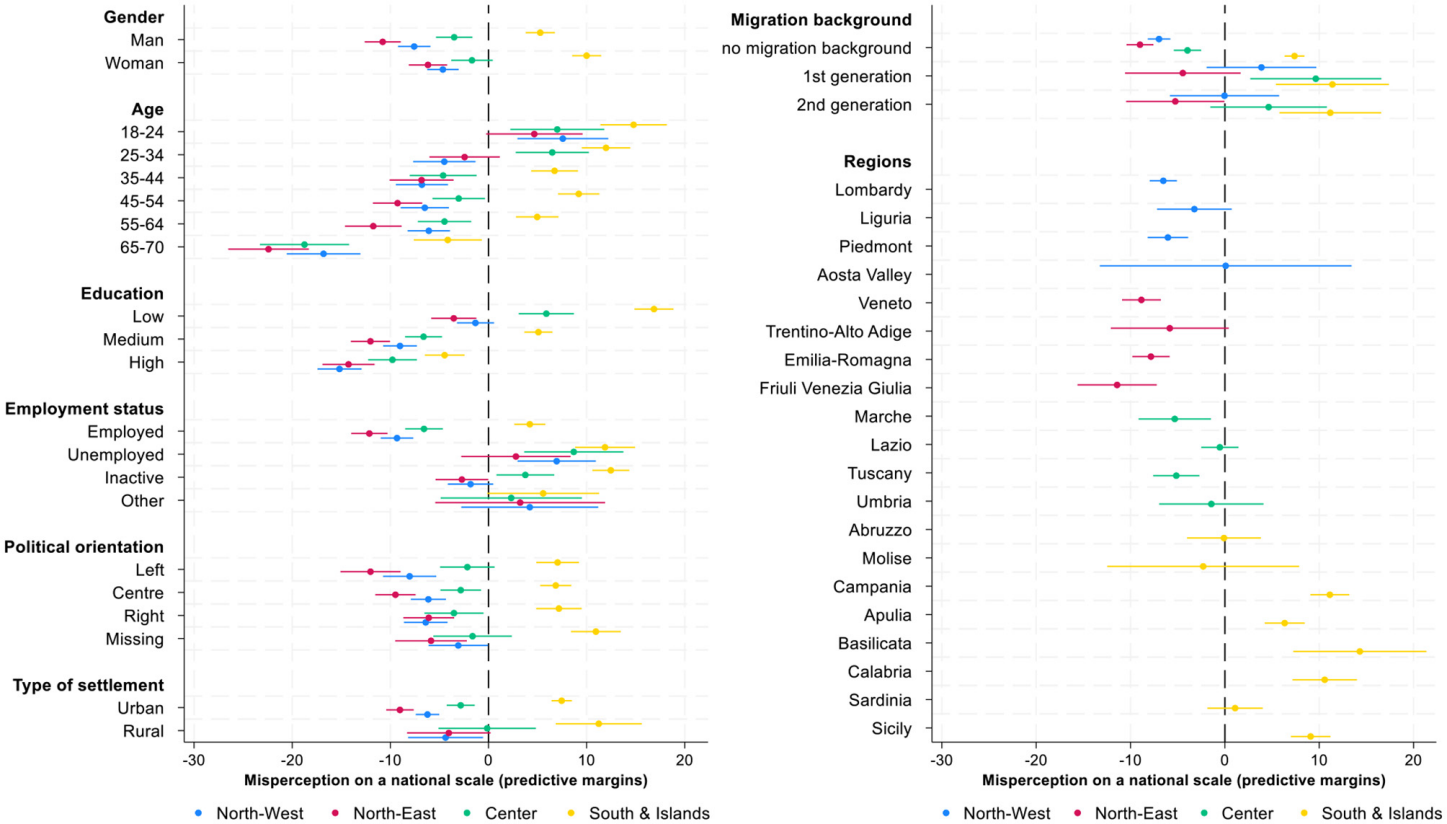
Predictive margins of individual and contextual correlates of income ranks misperception in national income distribution by macro area, 95% CI. Source: IneqPer 2024, Italy, *N* = 11,975.

**Figure 11:**
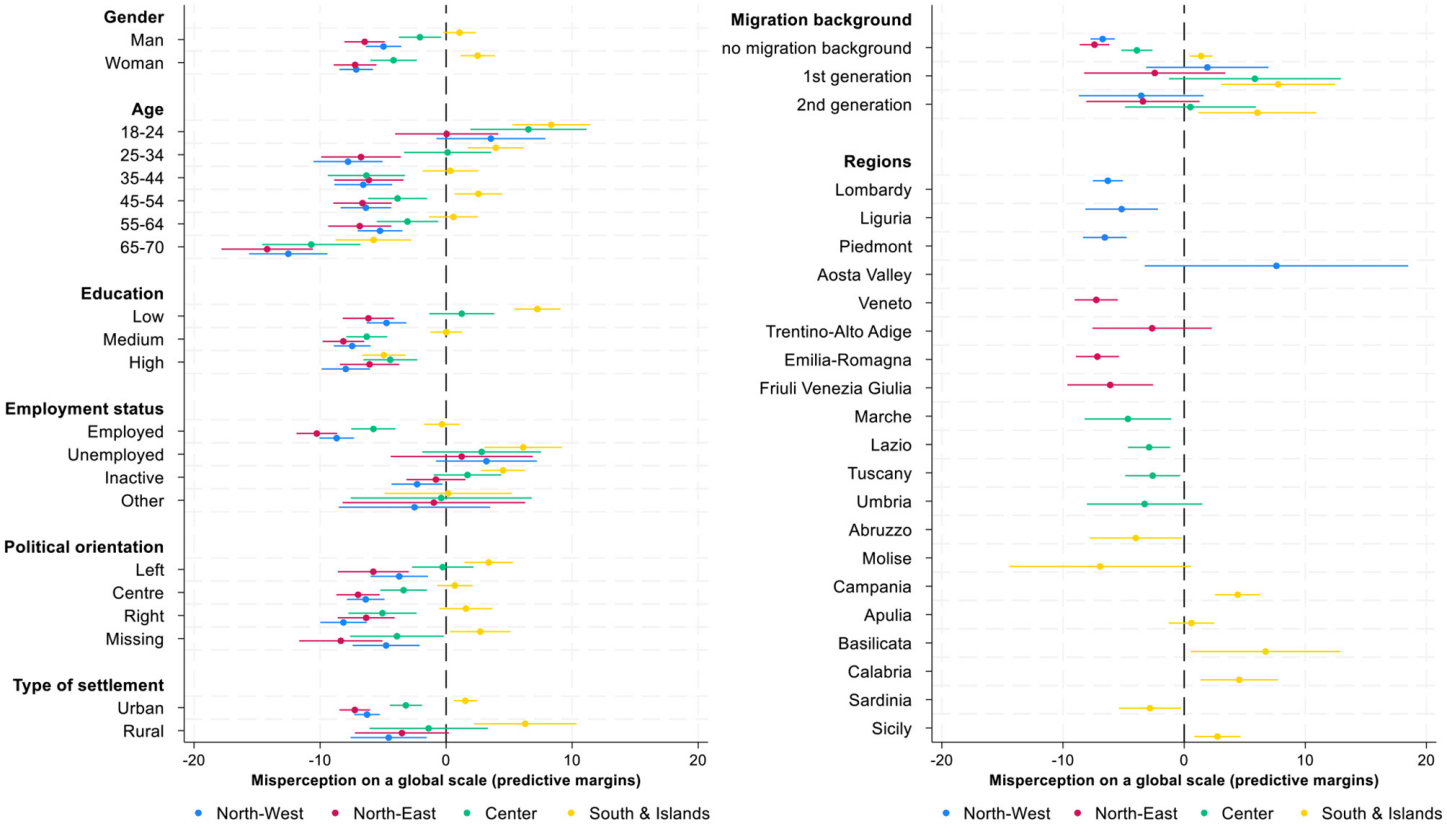
Predictive margins of individual and contextual correlates of income ranks misperception in global income distribution by macro area, 95% CI. Source: IneqPer 2024, Italy, *N* = 11,975.

Another layer of analyses concerns regional variation: the coefficients across macro-regions systematically differ for each and every dimension of interest beyond the dominant cleavage along South and North divide (Figures 10, 11). Some of the relevant outliers are respondents from rural Southern areas or low educated Southern respondents who tend to largely over-estimate their position in both national and global rankings.

## 7 Discussion and conclusions

This article is among the first studies to systematically describe (mis)perceptions of individual income ranking on national and global scales, understood as the gap between subjective and objective income positions on the income ladder. It is based on the most comprehensive national dataset (*n* = 12,000) on income perceptions collected in Italy to date (IneqPer project, 2024/2025). The study has two main goals: to describe how accurately Italians perceive their income rank both in national and global contexts and examine the individual and contextual factors that correlate with these perceptions. Another key contribution of the research is its emphasis on the concept of global interpersonal inequality, used to assess the discrepancies between individuals' actual and perceived positions in the global income distribution (Milanovic, 2016). This innovative angle aligns more closely with recent works by Fehr et al. (2022) and Nair (2018), which focus on perceptions of global inequality, rather than those by Sattler-Bublitz et al. (2024) and Ziano et al. (2024), which primarily address between-country inequality.

This article presents several key findings for the Italian context. First, the analysis confirms patterns observed in previous studies: individuals follow center tendency bias in their perceptions of relative income ranks. The data for Italy show that people frequently fail to accurately place themselves within both national and global income distributions: in all instances, they identify themselves as belonging to the middle part of the income distribution. Italians who have lower incomes systematically overestimate their income rank, while those with higher incomes underestimate it. This bias is revealed for perceptions of individual ranks in both national and global income distribution, underscoring a common pattern where individuals regress toward the perceived center. The findings are consistent with previous literature on developed countries, including Engelhardt and Wagener (2018), Fernández-Albertos and Kuo (2018), Fehr et al. (2022), and Sattler-Bublitz et al. (2024). Both global and national positional indicators point in the same direction, reflecting a general tendency among respondents to position themselves near the center of the income distribution.

Second, consistent patterns emerge regarding individual level correlates of misperceptions across all model specifications: younger individuals, unemployed, and immigrants tend to overestimate their income rank, whereas men, older individuals, those with higher levels of education, employed people, natives and those in urban areas are more prone to underestimation. The role of immigrant status is particularly interesting on the national rank: while first-generation immigrants exhibit the most pronounced misperceptions, subsequent generations show slight adjustments toward natives. Although the adjustment in rather limited in size, this might suggest a gradual adaptation of income perceptions over generations. A similar pattern is partially observed at the global level: first-generation immigrants tend to overestimate their income position, those without a migration background tend to underestimate it, and subsequent generations are relatively accurate in their estimates. Overall, there is a clear pattern that those who have strong social position and more resources of different kinds tend to underestimate their income rank while those who are more disadvantaged are inclined to overestimate their position on income ladder. Political orientation is also tested and while it shows no strong correlation with misperceptions of national income rank, individuals with left-leaning orientations tend to be more accurate in assessing their global income position, whereas those with center- or right-leaning orientations are more likely to underestimate it. This result is partly consistent with evidence from Germany, where ideology has been found to correlate with perceptions of national income ranking but only when ideology is decomposed from simple left-right classification: socially conservative individuals are especially prone to underestimating their relative position within the national income distribution (Busemeyer et al., 2024).

General results, however, are more nuanced and some categories of population are particularly prone to under or over estimation. This will be a great concern, for instance, for low-educated women and the low-educated young groups, respondents from rural Southern areas or low educated Southern respondents who are largely over-estimators. On the contrary, some groups tend to largely under-estimate their position like older generations of highly educated, although mostly with regard to national income rank.

Third, regional disparities highlight a significant role of geographical context on misperceptions. We observe a systematic overestimation of income rank in lower-income regions and underestimation in higher-income regions for both global and national misperceptions. The residents of more developed Northern regions are more likely to underestimate their income rank while the residents from less developed Southern regions tend to overestimate their position. These findings align with previous research on “median bias” or “center bias” at the macro-level and are consistent with the literature on between-country inequality that reports bias in rich areas of the world (Sattler-Bublitz et al., 2024).

Fourth, a distinctive contribution of this study is that it simultaneously assesses both national and global income misperceptions. This dual perspective allows for a deeper exploration of the underlying patterns of misperception, revealing a common logic across both levels. Although the more systematic comparison of the coefficients across models goes beyond the aim of the current analysis, the direction and magnitude of results remain rather consistent between the analysis of national and global distribution, an observation also reported by Fehr et al. (2022) in their study of Germany. Both income distributions seem to be perceived distant and abstract. This finding is particularly surprising, given the greater potential for misperception when considering income distribution at the global level. Overall, it appears that individuals seem to systematically project their perceived position within the national income distribution onto the global context. In part, this might be a result of “median” or “center” bias. However, as also signaled by Fehr et al. (2022), this pattern may as well reflect an anchoring effect: given the limited information available about the global context and cross-country comparisons, individuals tend to rely on their perceived national position as a starting point and make only partial adjustments from it. Moreover, they may consider their co-nationals a reference group and appear to extend this knowledge to the global level. As a result, people are comparably inaccurate in assessing their relative position in both contexts.

More broadly, both anchoring effects and reference group theory can provide complementing explanations to the cognitive biases behind misperceptions found in this study. They provide a general framework within which “center” bias can appear, as individuals compare themselves to their peers, family, nationals when making their own judgement and are exposed to different anchors and reference points Hvidberg et al., (2023).

We acknowledge some limitations of this research. First, although misperceptions may stem from different cognitive processes, they also depend on respondents' attention span during the survey and on potential misunderstandings of the questions (Enke and Graeber, 2023). While the tools employed in the Ineqper survey were designed to facilitate a smooth decision-making process (e.g., an easy-to-use continuous ranking slider), these factors should nonetheless be taken into account when interpreting the data and results. Second limitation concerns the construction of the objective global position within the global income distribution. In the absence of any better matched measures, the objective global position was calculated using the measure of post-tax national income in the WID dataset that includes also in kind-transfers, which are not included in the IneqPer data. This allows for some discrepancy and under-estimation of real individual position, although to a minor degree as the Italian welfare state does not heavily rely on non-cash transfers: for instance in 2023 in-kind benefits represented only one fifth of the total social protection benefits (Eurostat dataset, 2025). Finally, WID dataset is the only one freely available dataset with the information on individual incomes around the world. However, while the data are of high quality and the methodology is advanced, it is important to recognize some inherent limitations. Specifically, WID's data series and methodology are subject to ongoing revision, and due to limited data access in many countries, the series should be considered provisional.^8^ Differences may, therefore, arise when alternative measures of income are employed, such as post-tax disposable income, as well as when different anchoring methods are used. Future studies may also use other datasets such as the Luxembourg Income Study Database to compare and test the consistency of the findings.

Overall, the study highlights that income rank misperceptions are associated with both social factors and contextual conditions. The existence of such perception biases, and their systematic variation across groups, is important to acknowledge, as addressing them can contribute to a more accurate understanding of economic and political realities in Italy and beyond, within an increasingly complex global economy. Future research could explore their role for attitudes toward redistribution at both national and global levels, as well as attitudes to different political agendas and political behavior.

## Data Availability

The dataset generated and analyzed in this study are currently under institutional embargo and are not publicly available. Data access may be granted on a case-by-case basis for academic research purposes. Requests to access the data should be directed to the IneqPer PI at: https://ineqper.unipv.it/, and nevena.kulic@unipv.it.

## Footnotes

1 Sattler-Bublitz et al. (2024) examine the various terms used to describe the tendency of both poor and rich people to perceive themselves as middle class. They favor the term “central tendency of misperceptions,” but note other terms in the literature, including “center bias,” “median bias,” and “middle-class bias.” These are used inter-changeably in this manuscript.

2 WID provides data on an annual basis. To allow comparison with our monthly data, the annual values are divided by 12 to obtain monthly estimates.

3
https://wid.world/

4 The WID dataset presents post-tax income data in three formats: share of income (s), average income (a), and threshold (t). The thresholds are essential for constructing and comparing income rank distributions, as they identify the minimum and maximum income required to reach a given percentile.

5
https://wid.world/codes-dictionary/

6 The category rural includes settlements below 2000 inhabitants and farms and houses in the countryside, while the category urban includes a large city (>1 million inhabitants), a municipality near a large city, a city with 50,000 to 1 million inhabitants and a small town or urban village (less than 50,000 inhabitants).

7 Both White's test and Breusch–Pagan Test confirm the presence of heteroscedasticity. Shapiro–Wilk test signals that the residuals deviate from a normal distribution.

8
https://wid.world/document/distributional-national-accounts-guidelines-2020-concepts-and-methods-used-in-the-world-inequality-database/
