# Supplementary material for: (Mis)perceptions of individual position in national and global income distribution. The Italian case
Source: Front Sociol. 2026 Jan 14;10:1646173. doi: 10.3389/fsoc.2025.1646173 (PMC12848915; doi:10.3389/fsoc.2025.1646173)

Appendix

Table A1. Correlates of *misperception of income ranks in national income distribution: Predictive margins* for Figure 6, 95% CI

DEPENDENT VARIABLE	Misperception of income position in national income distribution
INDEPENDENT VARIABLE	Gender, age, education, employment, migration background, regions, political orientation, self-definition of residence (urban-rural)
Man	-2.98***<br>(0.431)
Woman	0.62<br>(0.445)
18-24	9.06***<br>(1.094)
25-34	3.89***<br>(0.796)
35-44	-1.62**<br>(0.727)
45-54	-1.04*<br>(0.618)
55-64	-3.14***<br>(0.618)
65-70	-14.20***<br>(1.002)
Low education	5.68***<br>(0.561)
Medium education	-4.40***<br>(0.439)
High education	-10.44***<br>(0.591)
Employed	-4.84***<br>(0.446)
Unemployed	7.49***<br>(1.048)
Inactive	4.15***<br>(0.598)
Other	3.36*<br>(1.779)
No migrant background	-1.90***<br>(0.318)
1st generation	6.72***<br>(1.616)
2nd generation	4.03***<br>(1.441)
Left	-2.39***<br>(0.672)
Center	-1.67***<br>(0.469)
Right	-1.16*<br>(0.639)
Missing	1.60**<br>(0.807)
Urban	-1.43***<br>(0.315)
Rural	1.72<br>(1.106)
Lombardy	-7.24***<br>(0.727)

Liguria	-3.63*
	(1.997)
Piedmont	-6.89***
	(1.088)
Aosta Valley	-0.57
	(6.938)
Veneto	-8.73***
	(1.054)
Trentino-Alto Adige	-6.01*
	(3.170)
Emilia-Romagna	-7.93***
	(1.002)
Friuli Venezia Giulia	-11.37***
	(2.128)
Marche	-3.94**
	(1.979)
Lazio	0.34
	(0.983)
Tuscany	-4.05***
	(1.255)
Umbria	-0.51
	(2.838)
Abruzzo	0.24
	(1.986)
Molise	-3.68
	(5.105)
Campania	10.71***
	(1.055)
Apulia	6.48***
	(1.084)
Basilicata	14.54***
	(3.654)
Calabria	10.15***
	(1.747)
Sardinia	1.78
	(1.507)
Sicily	9.08***
	(1.081)
Observations	11,975

Standard errors in parentheses
*** p<0.01, ** p<0.05, * p<0.1

Table A2. Correlates of *misperception of income ranks in global income distribution. Predictive margins* for Figure 7, 95% CI

DEPENDENT VARIABLE	Misperception of income position in global income distribution
INDEPENDENT VARIABLE	Gender, age, education, employment, migration background, regions, political orientation, self-definition of residence (urban-rural)
Man	-2.561***
	(0.375)
Woman	-3.346***
	(0.389)
18-24	4.879***
	(0.998)
25-34	-1.657**
	(0.715)
35-44	-3.979***

	(0.644)
45-54	-2.937***
	(0.538)
55-64	-3.139***
	(0.538)
65-70	-10.37***
	(0.859)
Low education	0.0419
	(0.499)
Medium education	-4.905***
	(0.376)
High education	-6.054***
	(0.510)
Employed	-5.757***
	(0.389)
Unemployed	3.497***
	(1.030)
Inactive	1.270**
	(0.538)
Other	-0.916
	(1.601)
No migrant background	-3.540***
	(0.279)
1st generation	3.973***
	(1.476)
2nd generation	0.683
	(1.282)
Left	-1.004*
	(0.588)
Center	-3.482***
	(0.406)
Right	-3.927***
	(0.559)
Missing	-2.698***
	(0.738)
Urban	-3.189***
	(0.277)
Rural	-0.280
	(0.966)
Lombardy	-6.429***
	(0.622)
Liguria	-4.968***
	(1.534)
Piedmont	-6.804***
	(0.916)
Aosta Valley	6.917
	(5.531)
Veneto	-7.141***
	(0.911)
Trentino-Alto Adige	-2.914
	(2.511)
Emilia-Romagna	-6.995***
	(0.900)
Friuli Venezia Giulia	-5.780***
	(1.777)
Marche	-4.178**
	(1.828)
Lazio	-2.707***
	(0.859)
Tuscany	-2.247*
	(1.166)
Umbria	-3.030

	(2.416)
Abruzzo	-4.035**
	(1.903)
Molise	-8.052**
	(3.810)
Campania	3.782***
	(0.955)
Apulia	0.642
	(0.957)
Basilicata	6.638**
	(3.173)
Calabria	4.035**
	(1.637)
Sardinia	-2.090
	(1.310)
Sicily	2.731***
	(0.979)
Observations	11,975

Standard errors in parentheses
*** p<0.01, ** p<0.05, * p<0.1

Table A3. Correlates of *misperception of income ranks in national income distribution. Predictive margins representing interactions between education and age, and education and gender* for Figure 8, 95% CI

DEPENDENT VARIABLES	Misperception of income position in national income distribution	Misperception of income position in national income distribution
Low education	5.61***	5.63***
	(0.569)	(0.561)
Medium education	-4.42***	-4.47***
	(0.439)	(0.440)
High education	-10.22***	-10.51***
	(0.596)	(0.592)
18-24	9.14***	9.19***
	(1.124)	(1.097)
25-34	3.43***	3.91***
	(0.854)	(0.796)
35-44	-1.37*	-1.63**
	(0.750)	(0.726)
45-54	-1.10*	-1.11*
	(0.613)	(0.618)
55-64	-3.04***	-3.12***
	(0.601)	(0.616)
65-70	-14.00***	-14.20***
	(0.998)	(1.003)
Low education # 18-24	13.09***	
	(2.051)	
Low education # 25-34	7.71***	
	(1.573)	
Low education # 35-44	7.47***	
	(1.339)	
Low education # 45-54	7.55***	
	(1.068)	

Low education # 55-64	3.30***	
	(0.959)	
Low education # 65-70	-7.81***	
	(1.636)	
Medium education # 18-24	6.78***	
	(1.338)	
Medium education # 25-34	2.36**	
	(1.183)	
Medium education # 35-44	-5.30***	
	(1.104)	
Medium education # 45-54	-5.99***	
	(0.874)	
Medium education # 55-64	-5.74***	
	(0.926)	
Medium education # 65-70	-17.62***	
	(1.279)	
High education # 18-24	4.68**	
	(2.048)	
High education # 25-34	-4.05***	
	(1.286)	
High education # 35-44	-13.74***	
	(1.124)	
High education # 45-54	-11.34***	
	(1.208)	
High education # 55-64	-12.14***	
	(1.376)	
High education # 65-70	-21.11***	
	(1.988)	
Man	-2.98***	-3.07***
	(0.431)	(0.435)
Woman	0.61	0.58
	(0.444)	(0.442)
Low education # Man		2.36***
		(0.781)
Low education # Woman		8.86***
		(0.782)
Medium education # Man		-5.48***
		(0.604)
Medium education # Woman		-3.46***
		(0.647)
High education # Man		-10.67***
		(0.793)
High education # Woman		-10.36***
		(0.845)
Employed	-4.78***	-4.79***
	(0.446)	(0.446)
Unemployed	7.52***	7.49***
	(1.045)	(1.048)
Inactive	4.06***	4.03***
	(0.599)	(0.600)
Other	3.19*	3.63**

	(1.764)	(1.778)
No migrant background	-1.93***	-1.91***
	(0.318)	(0.318)
1st generation	7.02***	6.71***
	(1.623)	(1.618)
2nd generation	4.14***	4.12***
	(1.439)	(1.439)
Left	-2.41***	-2.26***
	(0.671)	(0.672)
Centre	-1.66***	-1.70***
	(0.469)	(0.469)
Right	-1.12*	-1.14*
	(0.639)	(0.638)
Missing	1.55*	1.48*
	(0.807)	(0.807)
Urban	-1.42***	-1.42***
	(0.315)	(0.315)
Rural	1.72	1.68
	(1.103)	(1.105)
Lombardy	-7.25***	-7.23***
	(0.727)	(0.726)
Liguria	-3.73*	-3.41*
	(1.988)	(1.998)
Piedmont	-6.88***	-6.93***
	(1.090)	(1.087)
Aosta Valley	0.03	-0.68
	(6.805)	(6.952)
Veneto	-8.91***	-8.69***
	(1.053)	(1.053)
Trentino-Alto Adige	-5.89*	-6.05*
	(3.182)	(3.156)
Emilia-Romagna	-7.97***	-7.87***
	(0.998)	(1.000)
Friuli Venezia Giulia	-11.37***	-11.43***
	(2.129)	(2.124)
Marche	-4.05**	-3.96**
	(1.990)	(1.974)
Lazio	0.42	0.29
	(0.983)	(0.984)
Tuscany	-4.08***	-4.00***
	(1.256)	(1.252)
Umbria	-0.27	-0.50
	(2.821)	(2.837)
Abruzzo	0.30	0.37
	(1.988)	(1.975)
Molise	-3.93	-3.55
	(5.141)	(5.075)
Campania	10.67***	10.71***
	(1.057)	(1.054)
Apulia	6.56***	6.47***
	(1.081)	(1.086)

Basilicata	14.35***	14.28***
	(3.655)	(3.624)
Calabria	10.23***	10.08***
	(1.747)	(1.757)
Sardinia	1.95	1.65
	(1.496)	(1.508)
Sicily	9.10***	9.06***
	(1.082)	(1.081)
Observations	11,975	11,975

Standard errors in parentheses
*** p<0.01, ** p<0.05, * p<0.1

Table A4. Correlates of *misperception of income ranks in global income distribution. Predictive margins representing interactions between education and age, and education and gender* for Figure 9, 95% CI

DEPENDENT VARIABLES	Misperception of income position in global income distribution	Misperception of income position in global income distribution
Low education	0.116	-0.00270
	(0.507)	(0.499)
Medium education	-4.905***	-4.965***
	(0.375)	(0.377)
High education	-5.838***	-6.104***
	(0.519)	(0.511)
18-24	4.800***	4.983***
	(1.027)	(1.001)
25-34	-1.782**	-1.650**
	(0.778)	(0.715)
35-44	-3.786***	-3.989***
	(0.668)	(0.644)
45-54	-2.947***	-2.994***
	(0.533)	(0.536)
55-64	-2.857***	-3.122***
	(0.520)	(0.538)
65-70	-10.28***	-10.37***
	(0.856)	(0.860)
Low education # 18-24	9.455***	
	(1.882)	
Low education # 25-34	0.482	
	(1.466)	
Low education # 35-44	0.521	
	(1.223)	
Low education # 45-54	1.034	
	(0.929)	
Low education # 55-64	-1.211	
	(0.854)	
Low education # 65-70	-9.624***	
	(1.384)	
Medium education # 18-24	2.604**	

	(1.200)	
Medium education # 25-34	-3.240***	
	(1.030)	
Medium education # 35-44	-6.223***	
	(0.938)	
Medium education # 45-54	-5.899***	
	(0.747)	
Medium education # 55-64	-4.426***	
	(0.762)	
Medium education # 65-70	-11.08***	
	(1.104)	
High education # 18-24	-1.490	
	(1.899)	
High education # 25-34	-4.147***	
	(1.093)	
High education # 35-44	-8.886***	
	(0.944)	
High education # 45-54	-6.413***	
	(1.051)	
High education # 55-64	-3.667***	
	(1.175)	
High education # 65-70	-10.30***	
	(1.735)	
Man	-2.590***	-2.631***
	(0.374)	(0.379)
Woman	-3.317***	-3.379***
	(0.389)	(0.387)
Low education # Man		-0.817
		(0.690)
Low education # Woman		0.803
		(0.694)
Medium education # Man		-3.743***
		(0.509)
Medium education # Woman		-6.174***
		(0.563)
High education # Man		-4.625***
		(0.690)
High education # Woman		-7.566***
		(0.723)
Employed	-5.772***	-5.717***
	(0.388)	(0.389)
Unemployed	3.320***	3.498***
	(1.035)	(1.030)
Inactive	1.350**	1.173**
	(0.539)	(0.538)
Other	-1.208	-0.695
	(1.602)	(1.600)
No migrant background	-3.524***	-3.544***
	(0.279)	(0.279)
1st generation	3.808**	3.961***
	(1.483)	(1.480)

2nd generation	0.570	0.752
	(1.280)	(1.279)
Left	-1.028*	-0.897
	(0.588)	(0.589)
Centre	-3.453***	-3.501***
	(0.406)	(0.406)
Right	-3.876***	-3.914***
	(0.558)	(0.558)
Missing	-2.811***	-2.797***
	(0.739)	(0.738)
Urban	-3.185***	-3.187***
	(0.277)	(0.277)
Rural	-0.333	-0.304
	(0.964)	(0.965)
Lombardy	-6.423***	-6.423***
	(0.622)	(0.622)
Liguria	-5.124***	-4.783***
	(1.537)	(1.539)
Piedmont	-6.764***	-6.835***
	(0.917)	(0.918)
Aosta Valley	7.058	6.828
	(5.529)	(5.512)
Veneto	-7.198***	-7.110***
	(0.910)	(0.911)
Trentino-Alto Adige	-2.966	-2.982
	(2.509)	(2.510)
Emilia-Romagna	-6.971***	-6.936***
	(0.899)	(0.897)
Friuli Venezia Giulia	-5.670***	-5.832***
	(1.773)	(1.778)
Marche	-4.141**	-4.184**
	(1.834)	(1.831)
Lazio	-2.688***	-2.740***
	(0.859)	(0.861)
Tuscany	-2.289**	-2.212*
	(1.164)	(1.164)
Umbria	-3.181	-3.021
	(2.418)	(2.407)
Abruzzo	-3.944**	-3.950**
	(1.901)	(1.886)
Molise	-8.491**	-7.945**
	(3.865)	(3.805)
Campania	3.829***	3.788***
	(0.957)	(0.953)
Apulia	0.662	0.638
	(0.957)	(0.956)
Basilicata	6.421**	6.474**
	(3.173)	(3.146)
Calabria	4.022**	3.966**
	(1.638)	(1.644)
Sardinia	-2.047	-2.209*

	(1.312)	(1.309)
Sicily	2.698***	2.717***
	(0.978)	(0.980)
Observations	11,975	11,975
Standard errors in parentheses		
*** p<0.01, ** p<0.05, * p<0.1		

Table A5. Correlates of *misperception of income position in national income distribution,* OLS models with interaction terms for education and age, and education and gender, 95% CI

DEPENDENT VARIABLES	Misperception of income position in national income distribution	Misperception of income position in national income distribution
Medium education (Ref: Low education)	-6.31***	-7.85***
	(2.409)	(0.992)
High education (Ref: Low education)	-8.42***	-13.03***
	(2.885)	(1.129)
25-34 (Ref: 18-24)	-5.38**	-5.28***
	(2.558)	(1.356)
35-44 (Ref: 18-24)	-5.63**	-10.82***
	(2.443)	(1.345)
45-54 (Ref: 18-24)	-5.55**	-10.30***
	(2.301)	(1.289)
55-64 (Ref: 18-24)	-9.79***	-12.31***
	(2.242)	(1.267)
65-70 (Ref: 18-24)	-20.90***	-23.39***
	(2.559)	(1.411)
Medium education # 25-34	0.95	
	(3.079)	
Medium education # 35-44	-6.46**	
	(2.940)	
Medium education # 45-54	-7.22***	
	(2.750)	
Medium education # 55-64	-2.74	
	(2.729)	
Medium education # 65-70	-3.51	
	(3.096)	
High education # 25-34	-3.35	
	(3.482)	
High education # 35-44	-12.79***	
	(3.329)	
High education # 45-54	-10.47***	
	(3.266)	
High education # 55-64	-7.03**	
	(3.307)	
High education # 65-70	-4.89	
	(3.797)	
Woman (Ref: Man)	3.59***	6.50***
	(0.635)	(1.088)

Medium education # Woman		-4.48***
		(1.371)
High education # Woman		-6.19***
		(1.554)
Unemployed (Ref: Employed)	12.30***	12.27***
	(1.166)	(1.168)
Inactive (Ref: Employed)	8.84***	8.81***
	(0.816)	(0.818)
Other (Ref: Employed)	7.97***	8.42***
	(1.828)	(1.838)
1st generation (Ref: No migrant background)	8.95***	8.62***
	(1.666)	(1.659)
2nd generation (Ref: No migrant background)	6.07***	6.03***
	(1.485)	(1.485)
Centre (Ref: Left)	0.75	0.56
	(0.808)	(0.809)
Right (Ref: Left)	1.29	1.12
	(0.924)	(0.924)
Missing (Ref: Left)	3.96***	3.74***
	(1.072)	(1.074)
Rural (Ref: Urban)	3.14***	3.10***
	(1.154)	(1.156)
Liguria (Ref: Lombardy)	3.53*	3.82*
	(2.124)	(2.131)
Piedmont (Ref: Lombardy)	0.38	0.30
	(1.316)	(1.313)
Aosta Valley (Ref: Lombardy)	7.28	6.55
	(6.846)	(6.993)
Veneto (Ref: Lombardy)	-1.65	-1.47
	(1.283)	(1.283)
Trentino-Alto Adige (Ref: Lombardy)	1.36	1.18
	(3.270)	(3.244)
Emilia-Romagna (Ref: Lombardy)	-0.72	-0.64
	(1.239)	(1.240)
Friuli Venezia Giulia (Ref: Lombardy)	-4.12*	-4.21*
	(2.251)	(2.248)
Marche (Ref: Lombardy)	3.20	3.27
	(2.121)	(2.107)
Lazio (Ref: Lombardy)	7.67***	7.52***
	(1.225)	(1.224)
Tuscany (Ref: Lombardy)	3.18**	3.23**
	(1.458)	(1.453)
Umbria (Ref: Lombardy)	6.98**	6.73**
	(2.917)	(2.933)
Abruzzo (Ref: Lombardy)	7.55***	7.60***
	(2.117)	(2.104)
Molise (Ref: Lombardy)	3.33	3.67

	(5.193)	(5.128)
Campania (Ref: Lombardy)	17.93***	17.94***
	(1.289)	(1.284)
Apulia (Ref: Lombardy)	13.81***	13.70***
	(1.307)	(1.309)
Basilicata (Ref: Lombardy)	21.60***	21.51***
	(3.728)	(3.695)
Calabria (Ref: Lombardy)	17.49***	17.31***
	(1.893)	(1.902)
Sardinia (Ref: Lombardy)	9.20***	8.88***
	(1.667)	(1.677)
Sicily (Ref: Lombardy)	16.36***	16.29***
	(1.309)	(1.306)
Constant	-0.65	0.98
	(2.331)	(1.699)
Observations	11,975	11,975
R-squared	0.159	0.158

Standard errors in parentheses
*** p<0.01, ** p<0.05, * p<0.1

Table A6. Correlates of *misperception of income position in global income distribution,* OLS models with interaction terms for education and age, and education and gender, 95% CI

DEPENDENT VARIABLES	Misperception of income position in global income distribution	Misperception of income position in global income distribution
Medium education (Ref: Low education)	-6.851***	-2.926***
	(2.195)	(0.863)
High education (Ref: Low education)	-10.94***	-3.808***
	(2.666)	(0.991)
25-34 (Ref: 18-24)	-8.972***	-6.633***
	(2.366)	(1.233)
35-44 (Ref: 18-24)	-8.934***	-8.972***
	(2.242)	(1.218)
45-54 (Ref: 18-24)	-8.421***	-7.977***
	(2.093)	(1.165)
55-64 (Ref: 18-24)	-10.67***	-8.105***
	(2.050)	(1.144)
65-70 (Ref: 18-24)	-19.08***	-15.36***
	(2.282)	(1.252)
Medium education # 25-34	3.128	
	(2.807)	
Medium education # 35-44	0.107	
	(2.657)	
Medium education # 45-54	-0.0818	
	(2.474)	
Medium education # 55-64	3.636	
	(2.458)	
Medium education # 65-70	5.400**	

	(2.747)	
High education # 25-34	6.315**	
	(3.191)	
High education # 35-44	1.538	
	(3.043)	
High education # 45-54	3.497	
	(2.980)	
High education # 55-64	8.489***	
	(3.014)	
High education # 65-70	10.27***	
	(3.413)	
Woman (Ref: Man)	-0.727	1.620*
	(0.552)	(0.960)
Medium education # Woman		-4.050***
		(1.203)
High education # Woman		-4.560***
		(1.355)
Unemployed (Ref: Employed)	9.093***	9.214***
	(1.128)	(1.124)
Inactive (Ref: Employed)	7.122***	6.890***
	(0.727)	(0.727)
Other (Ref: Employed)	4.564***	5.022***
	(1.655)	(1.652)
1st generation (Ref: No migrant background)	7.332***	7.505***
	(1.520)	(1.516)
2nd generation (Ref: No migrant background)	4.094***	4.296***
	(1.321)	(1.319)
Centre (Ref: Left)	-2.425***	-2.604***
	(0.705)	(0.707)
Right (Ref: Left)	-2.848***	-3.016***
	(0.807)	(0.808)
Missing (Ref: Left)	-1.783*	-1.900**
	(0.965)	(0.966)
Rural (Ref: Urban)	2.852***	2.883***
	(1.010)	(1.011)
Liguria (Ref: Lombardy)	1.299	1.640
	(1.662)	(1.663)
Piedmont (Ref: Lombardy)	-0.341	-0.411
	(1.110)	(1.111)
Aosta Valley (Ref: Lombardy)	13.48**	13.25**
	(5.568)	(5.550)
Veneto (Ref: Lombardy)	-0.775	-0.687
	(1.106)	(1.106)
Trentino-Alto Adige (Ref: Lombardy)	3.457	3.441
	(2.589)	(2.590)
Emilia-Romagna (Ref: Lombardy)	-0.548	-0.513
	(1.096)	(1.095)

Friuli Venezia Giulia (Ref: Lombardy)	0.753	0.592
	(1.879)	(1.884)
Marche (Ref: Lombardy)	2.283	2.240
	(1.939)	(1.937)
Lazio (Ref: Lombardy)	3.735***	3.683***
	(1.063)	(1.064)
Tuscany (Ref: Lombardy)	4.134***	4.211***
	(1.326)	(1.326)
Umbria (Ref: Lombardy)	3.242	3.402
	(2.499)	(2.488)
Abruzzo (Ref: Lombardy)	2.479	2.473
	(2.000)	(1.986)
Molise (Ref: Lombardy)	-2.068	-1.522
	(3.916)	(3.855)
Campania (Ref: Lombardy)	10.25***	10.21***
	(1.149)	(1.144)
Apulia (Ref: Lombardy)	7.085***	7.061***
	(1.146)	(1.145)
Basilicata (Ref: Lombardy)	12.84***	12.90***
	(3.235)	(3.207)
Calabria (Ref: Lombardy)	10.45***	10.39***
	(1.755)	(1.759)
Sardinia (Ref: Lombardy)	4.376***	4.215***
	(1.455)	(1.452)
Sicily (Ref: Lombardy)	9.121***	9.141***
	(1.166)	(1.166)
Constant	4.666**	2.132
	(2.129)	(1.533)
Observations	11,975	11,975
R-squared	0.077	0.077

Standard errors in parentheses
*** p<0.01, ** p<0.05, * p<0.1

Table A7. Correlates *of misperceptions of income ranks in national income distribution* by macro-regions for Figure 10, OLS regression coefficients, 95% CI

DEPENDENT VARIABLE	North-West Misperception of income position in global income distribution	North-East Misperception of income position in global income distribution	Center Misperception of income position in global income distribution	South & Islands Misperception of income position in global income distribution
Woman (Ref: Man)	2.92**	4.61***	1.81	4.73***
	(1.190)	(1.393)	(1.473)	(1.116)
25-34 (Ref: 18-24)	-12.07***	-7.11**	-0.51	-2.81
	(2.879)	(3.082)	(3.097)	(2.142)
35-44 (Ref: 18-24)	-14.36***	-11.49***	-11.63***	-8.05***
	(2.777)	(3.066)	(3.058)	(2.163)
45-54 (Ref: 18-24)	-14.07***	-13.93***	-10.04***	-5.59***
	(2.738)	(2.874)	(2.863)	(2.072)

55-64 (Ref: 18-24)	-13.66***	-16.41***	-11.49***	-9.82***
	(2.612)	(2.928)	(2.848)	(2.059)
65-70 (Ref: 18-24)	-24.38***	-27.08***	-25.76***	-18.93***
	(2.898)	(3.116)	(3.208)	(2.342)
Medium education (Ref: Low education)	-7.69***	-8.49***	-12.51***	-11.78***
	(1.330)	(1.577)	(1.760)	(1.278)
High education (Ref: Low education)	-13.85***	-10.73***	-15.68***	-21.33***
	(1.583)	(1.875)	(1.983)	(1.529)
Unemployed (Ref: Employed)	16.27***	14.93***	15.26***	7.66***
	(2.260)	(3.052)	(2.833)	(1.792)
Inactive (Ref: Employed)	7.49***	9.43***	10.33***	8.25***
	(1.597)	(1.812)	(1.937)	(1.373)
Other (Ref: Employed)	13.53***	15.36***	8.89**	1.36
	(3.670)	(4.511)	(3.822)	(3.019)
1st generation (Ref: No migrant background)	10.86***	4.54	13.60***	4.01
	(3.046)	(3.236)	(3.654)	(3.119)
2nd generation (Ref: No migrant background)	6.94**	3.74	8.60***	3.79
	(3.033)	(2.781)	(3.272)	(2.816)
Centre (Ref: Left)	1.91	2.54	-0.68	-0.19
	(1.632)	(1.860)	(1.757)	(1.348)
Right (Ref: Left)	1.64	5.94***	-1.36	0.13
	(1.781)	(2.033)	(2.059)	(1.623)
Missing (Ref: Left)	4.96**	6.17**	0.53	3.90**
	(2.098)	(2.490)	(2.518)	(1.745)
Rural (Ref: Urban)	1.84	4.98**	2.70	3.78
	(2.046)	(2.307)	(2.660)	(2.308)
Liguria (Ref: Lombardy)	3.29			
	(2.146)			
Piedmont (Ref: Lombardy)	0.49			
	(1.322)			
Aosta Valley (Ref: Lombardy)	6.61			
	(6.855)			
Trentino-Alto Adige (Ref: Veneto)		3.01		
		(3.368)		
Emilia-Romagna (Ref: Veneto)		1.01		
		(1.454)		
Friuli Venezia Giulia (Ref: Veneto)		-2.57		
		(2.391)		
Lazio (Ref: Marche)			4.78**	
			(2.212)	
Tuscany (Ref: Marche)			0.16	
			(2.335)	
Umbria (Ref: Marche)			3.88	
			(3.429)	
Molise (Ref: Abruzzo)				-2.20
				(5.549)
Campania (Ref: Abruzzo)				11.23***
				(2.251)
Apulia (Ref: Abruzzo)				6.44***
				(2.275)
Basilicata (Ref: Abruzzo)				14.41***
				(4.112)
Calabria (Ref: Abruzzo)				10.68***
				(2.645)
Sardinia (Ref: Abruzzo)				1.18
				(2.496)
Sicily (Ref: Abruzzo)				9.19***
				(2.271)

Constant	3.86	-1.29	7.05*	9.22***
	(3.130)	(3.521)	(3.961)	(3.158)
Observations	3,268	2,237	2,378	4,092
R-squared	0.114	0.120	0.140	0.143

Robust standard errors in parentheses
*** p<0.01, ** p<0.05, * p<0.1

Table A8. Correlates of *misperceptions of income ranks in global income distribution by macro-regions* for Figure 11, OLS regression coefficients, 95% CI

	North-West	North-East	Center	South & Islands
DEPENDENT VARIABLE	Misperception of income position in global income distribution	Misperception of income position in global income distribution	Misperception of income position in global income distribution	Misperception of income position in global income distribution
Woman (Ref: Man)	-2.169**	-0.760	-2.100	1.435
	(0.998)	(1.207)	(1.295)	(0.988)
25-34 (Ref: 18-24)	-11.34***	-6.794***	-6.413**	-4.393**
	(2.626)	(2.625)	(2.982)	(1.935)
35-44 (Ref: 18-24)	-10.13***	-6.171**	-12.85***	-8.000***
	(2.543)	(2.585)	(2.899)	(1.965)
45-54 (Ref: 18-24)	-9.920***	-6.676***	-10.39***	-5.766***
	(2.490)	(2.445)	(2.736)	(1.874)
55-64 (Ref: 18-24)	-8.799***	-6.892***	-9.598***	-7.755***
	(2.392)	(2.458)	(2.679)	(1.872)
65-70 (Ref: 18-24)	-16.07***	-14.24***	-17.23***	-14.09***
	(2.610)	(2.664)	(2.827)	(2.090)
Medium education (Ref: Low education)	-2.727**	-1.987	-7.538***	-7.230***
	(1.111)	(1.357)	(1.573)	(1.161)
High education (Ref: Low education)	-3.228**	0.0929	-5.670***	-12.18***
	(1.327)	(1.664)	(1.765)	(1.359)
Unemployed (Ref: Employed)	11.88***	11.50***	8.588***	6.436***
	(2.194)	(3.036)	(2.638)	(1.749)
Inactive (Ref: Employed)	6.379***	9.453***	7.460***	4.841***
	(1.364)	(1.579)	(1.799)	(1.238)
Other (Ref: Employed)	6.175*	9.290**	5.376	0.483
	(3.154)	(3.796)	(3.779)	(2.683)
1st generation (Ref: No migrant background)	8.643***	4.974	9.742***	6.357**
	(2.638)	(3.058)	(3.705)	(2.472)
2nd generation (Ref: No migrant background)	3.185	3.999	4.418	4.641*
	(2.698)	(2.476)	(2.855)	(2.536)
Centre (Ref: Left)	-2.662*	-1.208	-3.105**	-2.684**
	(1.370)	(1.661)	(1.548)	(1.189)
Right (Ref: Left)	-4.427***	-0.565	-4.781***	-1.804
	(1.481)	(1.828)	(1.828)	(1.454)
Missing (Ref: Left)	-1.043	-2.574	-3.630	-0.662
	(1.807)	(2.263)	(2.308)	(1.610)
Rural (Ref: Urban)	1.714	3.740*	1.805	4.743**
	(1.635)	(2.015)	(2.508)	(2.141)
Liguria (Ref: Lombardy)	1.141			

	(1.650)			
Piedmont (Ref: Lombardy)	-0.252			
	(1.116)			
Aosta Valley (Ref: Lombardy)	13.92**			
	(5.591)			
Trentino-Alto Adige (Ref: Veneto)		4.611*		
		(2.683)		
Emilia-Romagna (Ref: Veneto)		0.0921		
		(1.284)		
Friuli Venezia Giulia (Ref: Veneto)		1.153		
		(2.021)		
Lazio (Ref: Marche)			1.751	
			(2.033)	
Tuscany (Ref: Marche)			2.041	
			(2.185)	
Umbria (Ref: Marche)			1.379	
			(3.049)	
Molise (Ref: Abruzzo)				-2.946
				(4.271)
Campania (Ref: Abruzzo)				8.410***
				(2.150)
Apulia (Ref: Abruzzo)				4.592**
				(2.161)
Basilicata (Ref: Abruzzo)				10.71***
				(3.688)
Calabria (Ref: Abruzzo)				8.536***
				(2.518)
Sardinia (Ref: Abruzzo)				1.169
				(2.337)
Sicily (Ref: Abruzzo)				6.738***
				(2.169)
Constant	4.896*	-2.642	9.377**	6.047**
	(2.874)	(3.079)	(3.765)	(2.943)
Observations	3,268	2,237	2,378	4,092
R-squared	0.062	0.050	0.072	0.078

Standard errors in parentheses
*** p<0.01, ** p<0.05, * p<0.1

Figure A1. The Distribution of *Misperceptions of Income Rank in National Income* Distribution

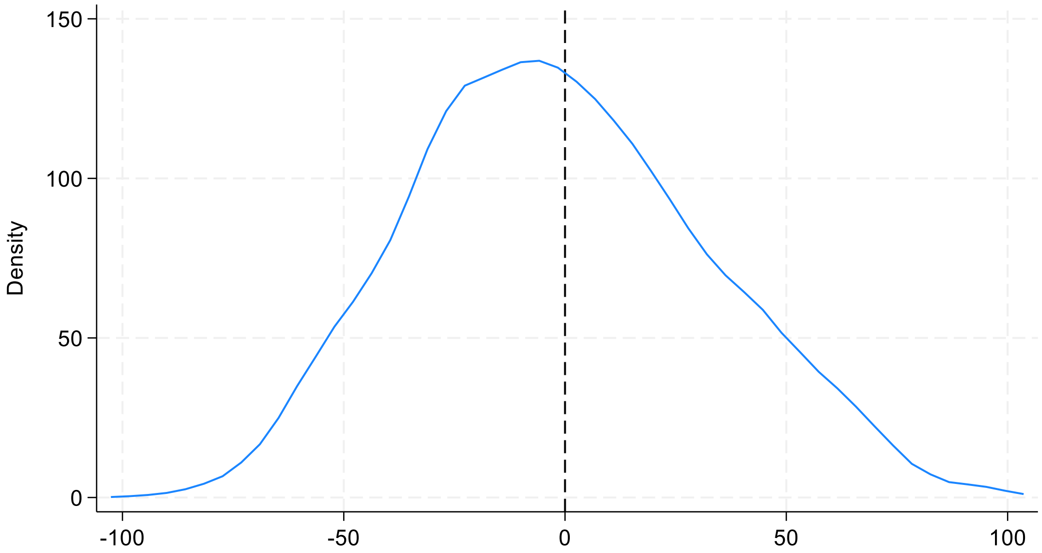

Figure A2. The Distribution of *Misperceptions of Income Rank in Global Income Distribution*

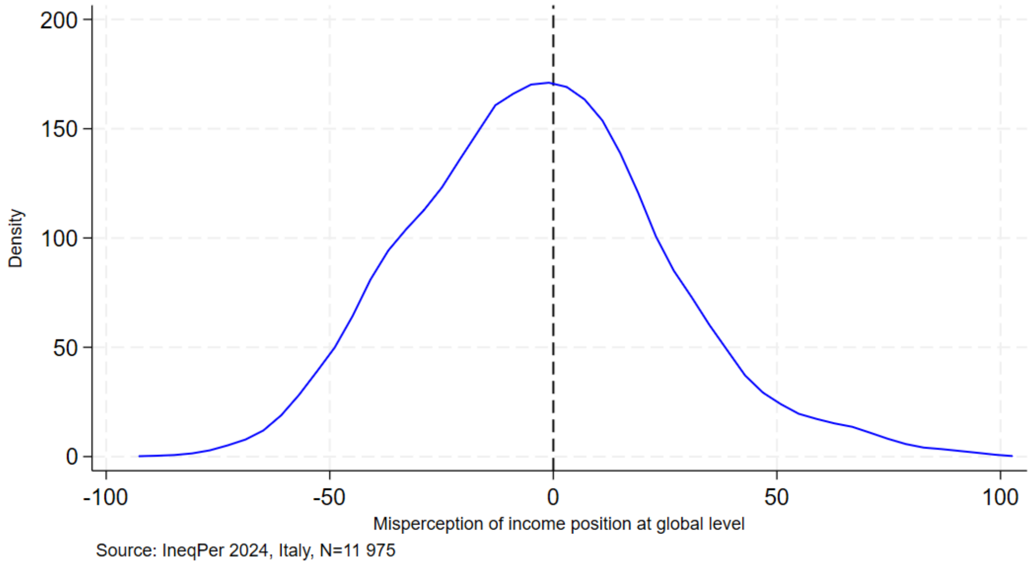

Supplement: Supplementary file 1 [file Data_Sheet_1.pdf]
